# Trastuzumab/pertuzumab combination therapy stimulates antitumor responses through complement-dependent cytotoxicity and phagocytosis

**DOI:** 10.1172/jci.insight.155636

**Published:** 2022-03-22

**Authors:** Li-Chung Tsao, Erika J. Crosby, Timothy N. Trotter, Junping Wei, Tao Wang, Xiao Yang, Amanda N. Summers, Gangjun Lei, Christopher A. Rabiola, Lewis A. Chodosh, William J. Muller, Herbert Kim Lyerly, Zachary C. Hartman

**Affiliations:** 1 Department of Surgery, Duke University, Durham, North Carolina, USA.; 2 Department of Cancer Biology, University of Pennsylvania (UPenn), Philadelphia, Pennsylvania, USA.; 3 Department of Biochemistry, McGill University, Montreal, Quebec, Canada.; 4 Department of Immunology and; 5 Department of Pathology, Duke University, Durham, North Carolina, USA.

**Keywords:** Oncology, Therapeutics, Cancer immunotherapy, Complement

## Abstract

Two HER2-specific mAbs, trastuzumab and pertuzumab (T+P), combined with chemotherapy comprise standard-of-care treatment for advanced HER2^+^ breast cancers (BC). While this antibody combination is highly effective, its synergistic mechanism-of-action (MOA) remains incompletely understood. Past studies have suggested that the synergy underlying this combination occurs through the different mechanisms elicited by these antibodies, with pertuzumab suppressing HER2 heterodimerization and trastuzumab inducing antitumor immunity. However, in vivo evidence for this synergy is lacking. In this study, we found that the therapeutic efficacy elicited by their combination occurs through their joint ability to activate the classical complement pathway, resulting in both complement-dependent cytotoxicity and complement-dependent cellular phagocytosis of HER2^+^ tumors. We also demonstrate that tumor C1q expression is positively associated with survival outcome in HER2^+^ BC patients and that complement regulators CD55 and CD59 were inversely correlated with outcome, suggesting the clinical importance of complement activity. Accordingly, inhibition of C1q in mice abolished the synergistic therapeutic activity of T+P therapy, whereas knockdown of CD55 and CD59 expression enhanced T+P efficacy. In summary, our study identifies classical complement activation as a significant antitumor MOA for T+P therapy that may be functionally enhanced to potentially augment clinical therapeutic efficacy.

## Graphical abstract

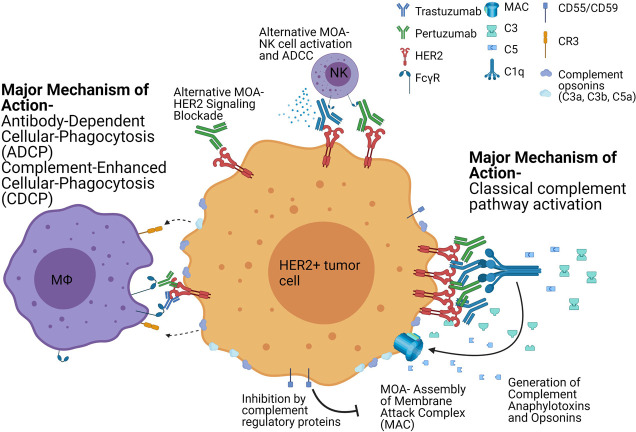

## Introduction

Approximately 20% of breast cancers (BC) have amplified expression of HER2, an oncogenic ErbB family member transmembrane protein recognized as a driver for an aggressive cancer phenotype (1, 2). Clinical outcomes for this disease have improved substantially with the advent and use of targeted HER2 mAb therapies, beginning with trastuzumab (Herceptin), which was approved in 1998 (3). Although trastuzumab treatment in combination with chemotherapy had positive outcomes, disease progression and recurrence continued to occur, especially in more advanced metastatic BC (4). This led to the 2012 approval of another humanized antibody targeting HER2, pertuzumab (Perjeta), used in conjunction with trastuzumab (5). Pertuzumab administration with trastuzumab and docetaxel improved the pathologic complete response (pCR) in the neoadjuvant setting (6). Likewise in the adjuvant setting, pertuzumab has been shown to improve disease-free survival (DFS) among patients with HER2^+^, operable BC when added to trastuzumab and chemotherapy (7). Thus, the combination of trastuzumab and pertuzumab (T+P) with chemotherapy has become the standard-of-care treatment for most newly diagnosed HER2^+^ BCs (8–10). However, while this combination has demonstrated efficacy, it is still unclear how pertuzumab synergizes with trastuzumab to elicit such a significant benefit in efficacy, as they both target HER2, albeit at different regions (11). Early studies demonstrated that pertuzumab binds to the center of domain II of HER2, allowing for steric inhibition of HER2 heterodimerization with HER3/EGFR (12, 13), which impairs HER2 oncogenic signaling in vitro (14, 15). In preclinical HER2^+^ xenograft models, pertuzumab was shown to strongly enhance antitumor activity when combined with trastuzumab, which was thought to be linked to its additional inhibition of HER2 signaling (13, 16, 17). However, this antitumor efficacy could also be due to the activation of other innate immune mechanisms of action, which to date, remain unexplored for pertuzumab and for T+P combinations. As these therapies are not always curative, especially in Stage IV HER2^+^ BC, it is essential to understand their mechanisms of action as a means to improve them and identify biomarkers for therapy resistance (11).

Tumor-targeting therapeutic mAbs (e.g., T+P) of the human IgG1 isotype have the capacity to elicit multiple mechanisms of antitumor action (11, 18, 19). These include (a) direct antitumor activities (e.g., antiproliferation and apoptosis induction), (b) indirect antitumor activities through engagement with Fcγ receptors (FCGRs) on immune cells to induce antibody-dependent cellular cytotoxicity (ADCC) or antibody-dependent cellular phagocytosis (ADCP), or (c) activation of complement cascades resulting in complement-dependent cytotoxicity (CDC) or complement-dependent cellular phagocytosis (CDCP). The contribution of each mechanism to the therapeutic efficacy demonstrated by T+P in clinical settings remains unclear. In this study, we investigated the in vivo mechanism of action (MOA) of pertuzumab in combination with trastuzumab in various HER2^+^ BC preclinical models. We previously reported ADCP by macrophages as the dominant role of trastuzumab-induced antitumor efficacy and its synergy with ADCP-enhancing CD47 checkpoint blockade (20). In this study, we found that the addition of pertuzumab to trastuzumab therapy synergistically elicits activation of the classical complement pathway in vivo and is critical for the enhanced antitumor efficacy of this combination therapy.

The complement system consists of more than 50 serum proteins and membrane-bound regulators/receptors, which can be locally produced by multiple cell types, including macrophages, fibroblasts, and tumor cells (21). Activation of the complement cascade induces diverse effector functions, including cell lysis, phagocytosis, chemotaxis, and immune cell activation (22–25). Antibodies can activate the classical complement pathway when bound to cells through C1q binding, triggering a cascade of enzymatic cleavage reactions to form the C3 convertase (26, 27). The cleavage of the central component C3 leads to formation of C3b and its deposition on the targeted cell membrane. The converted forms of C3b serve as important opsonins that can boost immune effector cell functions upon binding to complement receptors (such as CR1, CR3) on macrophages and NK cells (22, 23, 28, 29). Additionally, in the complement cascade, the deposition of membrane-bound C3b interacts with C3 convertase to form C5 convertase, which consequently cleaves C5 and activates the terminal pathway, leading to deposition of the membrane-attack complex (MAC). This complex will directly lyse the target cell, a process called CDC.

Activation of the entire complement cascade is highly regulated by membrane-bound complement regulatory proteins (CRPs) that serve to prevent severe pathology resulting from aberrant complement activation. Both CD55 (decay-accelerating factor [DAF]) and CD59 (protectin) are GPI-anchored CRPs often found upregulated in cancer cells, and they can be expressed either on cell surface or secreted in a soluble form in the tumor microenvironment (29, 30). CD55 accelerates the decay of C3 and C5 convertase, whereas CD59 (protectin) prevents the polymerization of C9, resulting in inhibition of MAC assembly and cell lysis.

In the current study, we demonstrate that, while pertuzumab has different MOAs, only the T+P combination allows for activation of the classical complement pathway. Moreover, we found that this pathway is critical for the emergent antitumor efficacy of the T+P combination, which could be enhanced by targeting complement regulators, suggesting a potentially novel clinical means of enhancing antitumor mAb therapies.

## Results

### Synergistic antitumor therapeutic effects of T+P in vivo.

**Figure 1:**
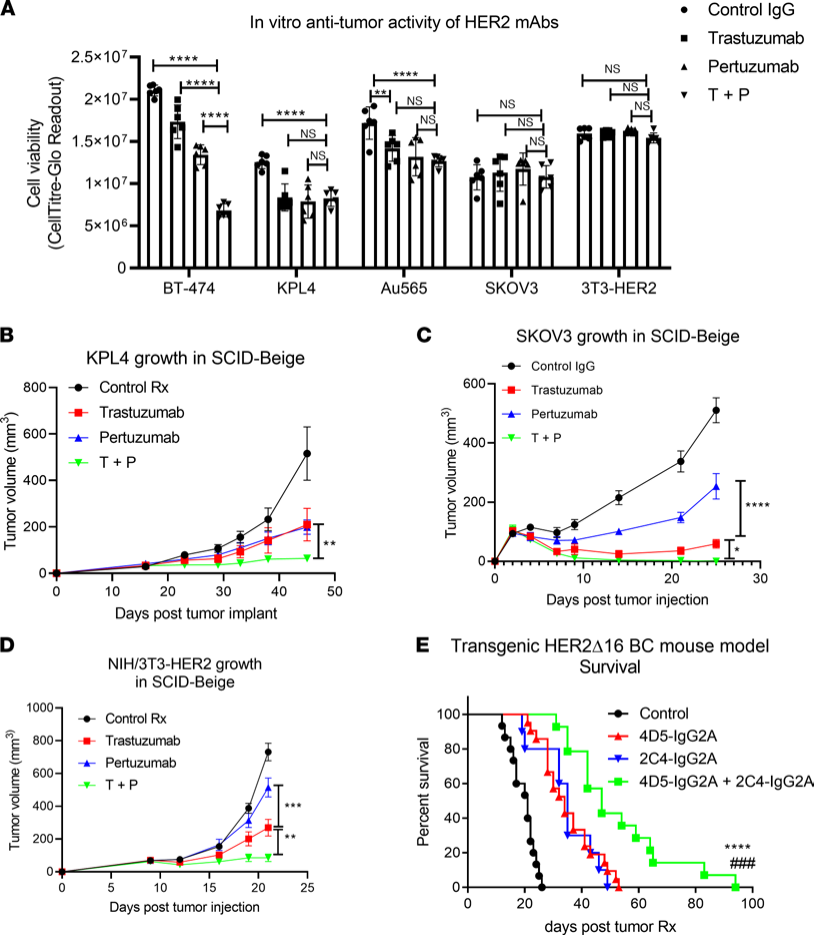
Synergistic therapeutic antitumor activity of T+P combination in vivo. (**A**) HER2^+^ tumor model cell-lines (KPL-4, Au565, SKOV3, BT-474, 3T3-HER2) were treated with 20 μg/mL of HER2 mAbs, as indicated, and cultured in vitro for 6 days. Cell viability were analyzed by CellTiter-Glo assay. *n* = 6. (**B**) KPL-4 cells were implanted into mammary fat pads of SCID-beige Balb/c mice (5 × 10^5^ cells). HER2 mAbs (100 μg each) or control human IgG1 were administered weekly starting on day 21, and tumor volume was measured. *n* = 10. (**C**) SKOV3 (HER2^+^/HER3^–^ ovarian cancer line) were implanted into the flank of SCID-beige Balb/c mice (1 × 10^6^ cells each). HER2 mAbs (100 μg each) were administered weekly. *n* = 5. (**D**) NIH/3T3 cells stably expressing HER2 were implanted into the flank of SCID-beige Balb/c mice (2 × 10^5^ cells). HER2 mAbs (200 μg each) or control human IgG1 were administered weekly. *n* = 5. (**A**–**D**) Data are shown as mean ± SEM. Two-way ANOVA with Tukey’s multiple-comparison post hoc test; *P < 0.05, **P < 0.01, ***P < 0.001, ****P < 0.0001. (**E**) Experiment using an immunocompetent, human HER2Δ16 transgenic mouse model treatment with murinized HER2 mAbs. Spontaneous breast tumors in the transgenic mice were induced with doxycycline diet. Four treatment arms were set up: Control mouse IgG (200 μg weekly, *n* = 15), 4D5-IgG2A (murinized Trastuzumab, 200 μg weekly, *n* = 16), 2C4-IgG2A (murinized Pertuzumab, 200 μg weekly, *n* = 10), and 4D5-IgG2A combined with 2C4-IgG2A (*n* = 14). Individual animals were consecutively enrolled into a specific treatment arm as soon as palpable breast tumors were detected (~100 mm^3^). Day 0 is the day of palpable tumor detection and treatment enrollment. Log-rank (Mantel-Cox) test for survival analysis, ****P < 0.0001 of treatment group vs control group, ^##^*P* < 0.01 significant difference observed between 4D5-IgG2A and 4D5-IgG2A + 2C4-IgG2A groups.

To understand the effect and mechanisms underlying T+P in HER2^+^ cancers, we first tested their potential synergy in different HER2^+^ cancer models both in vitro and in vivo. As multiple reports have indicated that pertuzumab inhibits tumor growth through blockade of HER2/HER3 dimerization and downstream oncogenic signaling, we speculated that synergy would be observed against HER2/HER3 expressing lines both in vitro and in vivo, while this synergy would not be observed in HER2^+^HER3^–^ lines (16, 17). We found that, while these HER2-specific mAbs demonstrated a synergistic effect against the growth of BT-474 cells in vitro, consistent with a previous report (14), this synergy was not seen in HER2^+^/HER3^+^ BC models such as KPL-4 and Au565 cells, or in HER2^+^/HER3^–^ models, such as SKOV3 (31) or NIH/3T3-expressing HER2 in vitro (Figure 1A). Expression (or absence) of HER2, HER3, and EGFR in these cell lines has been confirmed by different methods (Supplemental Figures 1 and 2; supplemental material available online with this article; https://doi.org/10.1172/jci.insight.155636DS1). HER2 and HER3 phosphorylation were also present in these cells (Supplemental Figure 3), which led us to test the effect of HER2 mAbs on HER2/HER3 oncogenic signaling inhibition. Our in vitro analysis confirmed the ability of pertuzumab to block HER2/HER3 heterodimer–mediated signaling, but the combination with trastuzumab did not further enhance this signaling blockade (Supplemental Figure 4, A and B). In contrast to in vitro tumor cell growth, when KPL-4, SKOV3, and 3T3-HER2 cells were implanted into SCID-beige mice and treated in vivo, we consistently observed enhanced therapeutic efficacy with the combination therapy (T+P) compared with the monotherapy of each HER2 mAb (Figure 1, B–D). Of note, pertuzumab monotherapy was also less effective than trastuzumab in SKOV3 (Figure 1C) and 3T3-HER2 models (Figure 1D), reflective of its reduced efficacy as a monotherapy in a clinical setting (32). To determine if this synergy occurs in an immunocompetent context, we generated murinized pertuzumab (2C4-IgG2A) and trastuzumab (4D5-IgG2A) with the mouse IgG2A isotype, which is functionally equivalent to human IgG1, as we had previously reported in our study of trastuzumab (20). These murinized versions allowed for prolonged treatment of immunocompetent mice without the development of anti–human IgG responses to the antibodies themselves (33). The immunocompetent model we used develops spontaneous HER2^+^ BC that is driven by expression of a HER2Δ16 oncogene, without the expression of HER3. Notably, the oncogenic signaling driven by HER2Δ16 homodimer could not be effectively blocked by pertuzumab (Supplemental Figure 4, C and D). Using this model, we found that the addition of 2C4-IgG2A to 4D5-IgG2A significantly prolonged the survival of the tumor-bearing mice, in comparison with each mAb as a single agent. These sets of experiments revealed that, while both trastuzumab and pertuzumab could inhibit tumor growth on their own in vivo, the combination resulted in an enhanced therapeutic outcome often not observed on in vitro tumor growth and independent of HER2 signaling blockade. Our findings in adaptive immunodeficient SCID mice suggest an innate immune MOA that was an emergent property of this antibody combination.

### Role of Fc-FCGR engagement in HER2 mAbs antitumor activity.

**Figure 2:**
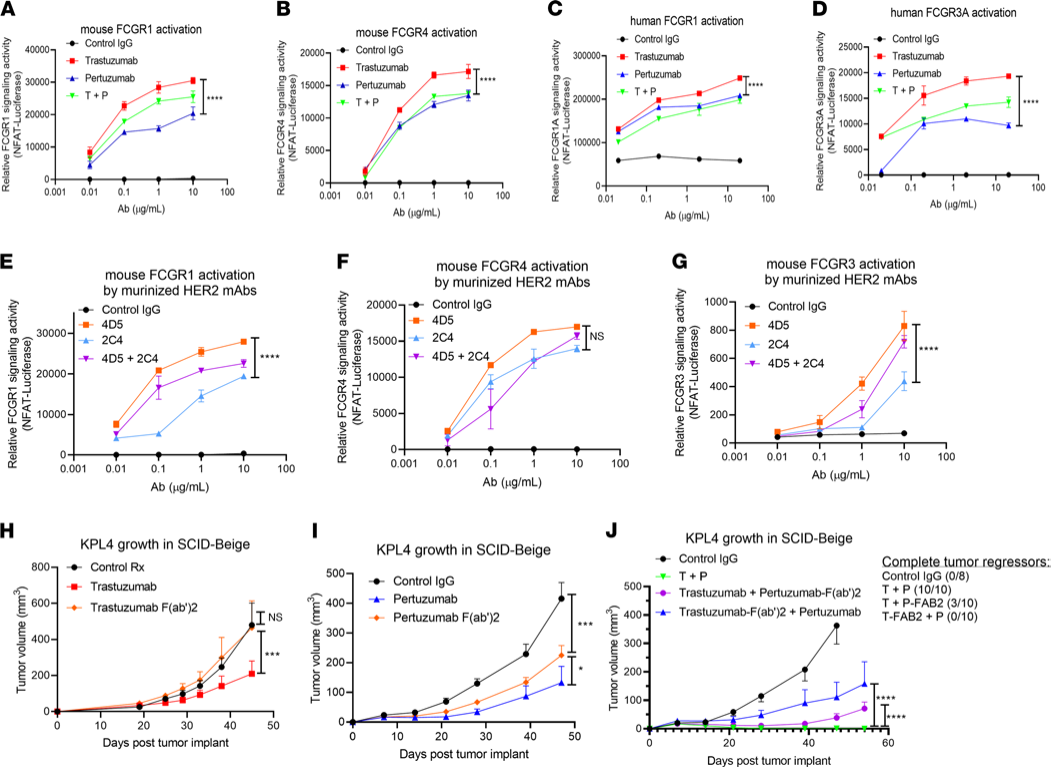
Role of FCGR engagements in HER2 mAb antitumor activity. (**A** and **B**) Mouse FCGR signaling activation assay. KPL-4 cells were plated and treated with indicated HER2 mAbs concentrations for 1 hour. Jurkat cells containing NFAT-luciferase reporter and expressing mouse FCGR1 (**A**) and FCGR4 (**B**) were added to the KPL-4 cells containing antibodies and cocultured for 4 hours. FCGR signaling activation were assessed by luciferase activity quantification. (**C** and **D**) Human FCGR signaling activation was similarly quantified with Jurkat-NFAT-Luciferase reporter cells expressing human FCGR1 (**C**) and FCGR3A (**D**). (**E**–**G**) Mouse FCGR signaling activation assays were repeated using murinized HER2 mAbs with the IgG2A isotype. In addition to mFCGR1 (**E**) and mFCGR4 (**F**), mouse FCGR3 (**G**) was tested here since mFCGR3 can be activated by murine antibodies but not human antibodies. All data represents mean ± SEM; *n* = 4. (**H**–**J**) Antigen-binding-fragment F(ab’)_2_ of the HER2 mAbs were generated and their therapeutic efficacy against HER2^+^ BC in vivo were compared with the parental antibody, respectively. As described before, KPL-4 cells (5 × 10^5^ cells) were implanted in mammary fat pads of SCID-beige mice and treated with the indicated HER2 mAbs or F(ab’)_2_ (100 μg per week). (**H**) Comparison between Trastuzumab versus Trastuzumab-F(ab’)_2_. (**I**) Comparison between Pertuzumab versus Pertuzumab-F(ab’)_2_. (**J**) Comparison between T+P versus Trastuzumab + Pertuzumab-F(ab’)_2_ versus Trastuzumab-F(ab’)_2_ + Pertuzumab. Numbers of mice showing total tumor regression are displayed on the right of the graph. (**H**–**J**) *n* = 8–10 for all groups. Two-way ANOVA with Tukey’s multiple-comparison post hoc test. All data represent mean ± SEM; *P < 0.05, ***P < 0.001, ****P < 0.0001.

Consistent with our previous study that demonstrated trastuzumab mediated ADCP through the activation of FCGRs, our data indicate that the T+P MOA likely includes an innate immune component. We next evaluated the ability of T+P to activate FCGR signaling using our previously described FCGR reporter assay (20, 34). In these studies, we tested both mouse and human activating FCGRs (i.e., mFCGR1, mFCGR3, mFCGR4, hFCGR1, hFCGR3A), which are expressed on macrophages to mediate ADCP and on NK cells to mediate ADCC (35, 36). We found weaker engagement with both mouse and human FCGRs by pertuzumab compared trastuzumab, and we found no synergistic increase in FCGR activation when the 2 antibodies were combined (Figure 2, A–D). These experiments were repeated using the murinized mAbs 4D5-IgG2A and 2C4-IgG2A, and similar results were observed (Figure 2, E–G). To assess the importance of the Fc-FCGR engagement of this therapy in vivo, we generated F(ab’)_2_ fragments of T+P through pepsin digestion (Supplemental Figure 5A), therefore blocking their ability to engage with extracellular receptors, such as FCGRs (Supplemental Figure 5B), while retaining their HER2 binding/blocking function (Supplemental Figure 5C). The in vivo antitumor activity of each F(ab’)_2_ was first compared with its parental mAb. In agreement with our previously published findings (20), we found trastuzumab activity to be strongly dependent on an intact Fc region, confirming the importance of FCGR engagement (Figure 2H). In contrast, pertuzumab F(ab’)_2_ retained partial antitumor activity compared with its parental mAb (Figure 2I). This suggests that pertuzumab monotherapy has the capacity to inhibit tumor growth in vivo through both Fc-mediated and Fc-independent mechanisms, such as HER2/HER3 signaling blockade. However, in a combination setting, we found that the presence of both Fc regions were essential for maximal therapeutic efficacy. The intact HER2 mAbs combination (T+P) completely prevented KPL-4 growth in all mice (10 of 10) but had a partial effect when the Fc region of pertuzumab was deleted (3 of 10 mice) and had an even further reduced efficacy when the Fc region of trastuzumab was deleted (0 of 10 mice, Figure 2J). Taken together, our in vitro and in vivo studies point to a synergistic MOA for T+P combination therapy that requires the Fc regions of both mAbs, but it is independent of enhanced FCGR activation. As the Fc region of antibodies are known to activate the classical complement pathway, leading to complement dependent cytotoxicity and phagocytosis (28, 29, 37), we next investigated the ability of these mAbs to elicit complement cascade activation.

### T+P combination therapy promotes complement activation, tumor opsonization, and CDC in vitro and in vivo.

**Figure 3:**
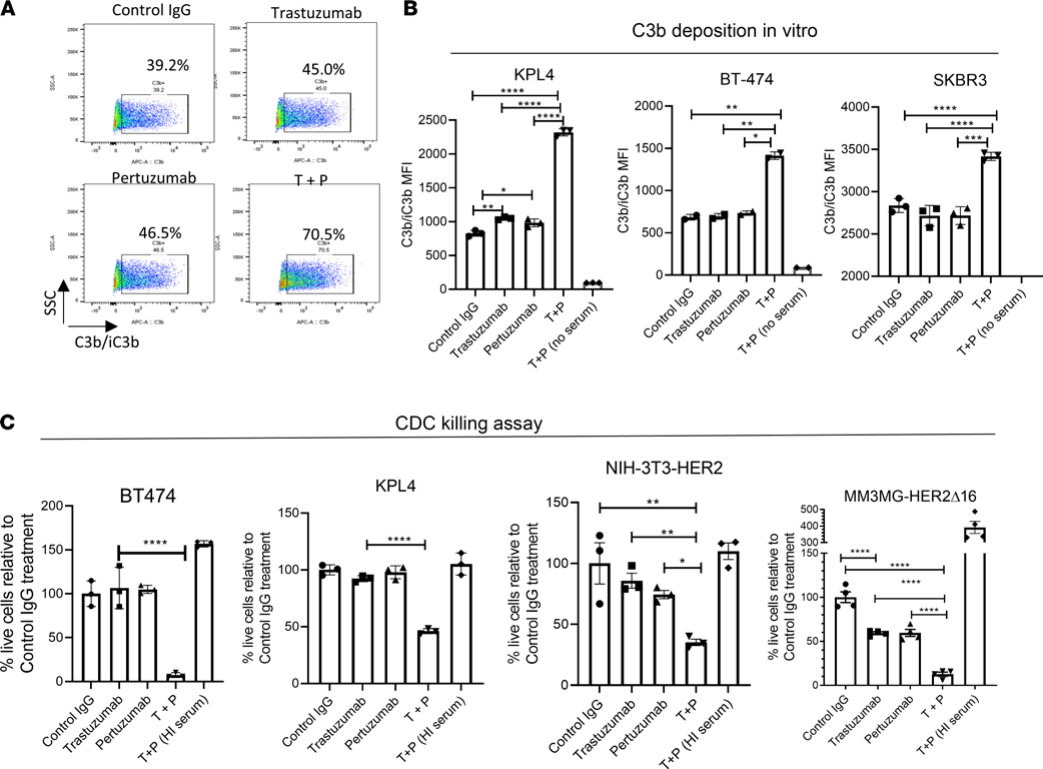
T+P combination therapy mediates complement activation, tumor cell opsonization, and complement-dependent cytotoxicity in vitro. (**A**) Representative FACS quantification plots of human complement 3b (C3b) surface deposition on KPL-4 cells after 30 minutes of incubation with normal human serum with indicated HER2 mAbs. (**B**) Graphical summary of C3b depositions staining described in **A**, confirmed on several HER2^+^ BC lines: KPL-4, BT-474, and SKBR3. *n* = 3; 1-way ANOVA with Tukey’s multiple-comparison post hoc test. Assay were repeated using normal human serum from 3 different donor sources. (**C**) Complement-dependent cytotoxicity (CDC) killing assays were performed on HER2^+^ cell lines in vitro (BT-474, KPL-4, NIH-3T3-HER2, MM3MG-HER2Δ16). Tumor cells were incubated with the indicated HER2 mAbs and with 25% of non-heat-inactivated normal rabbit serum. Cell viability were analyzed 2–4 hours after serum incubation, using CellTiter-Glo luminescent assay. Heat-inactivated (HI) serum was used as negative control. *n* = 3–4; 1-way ANOVA with Tukey’s multiple-comparison post hoc test. All data represent mean ± SEM; *P < 0.05, **P < 0.01, ***P < 0.001, ****P < 0.0001.

To determine the potential role of complement as an antitumor mechanism in HER2 mAb therapies, we first assessed the direct activation of complement in vitro by trastuzumab, pertuzumab, or their combination against HER2^+^ tumor cells. In these assays, we incubated HER2^+^ cell lines (KPL-4, BT-474, and SKBR3) with HER2 mAbs in the presence of non-heat-inactivated normal human serum (NHS) containing intact complement proteins. Tumor cell opsonization was measured by staining for C3b/iC3b deposition on the cell surface (Figure 3A). Strikingly, we found that the T+P combination therapy strongly and consistently increased tumor cell opsonization by C3b across multiple cell lines tested (Figure 3, A and B). Expectedly, this phenomenon was not seen when C1q was predepleted in the serum (Supplemental Figure 6A), indicating mAb activation of the classical complement pathway. Of note, single mAb treatments did not induce significant complement deposition, consistent with previous studies (20, 37).

Having demonstrated classical complement activation, we next determined if T+P-mediated complement activation leads to CDC of tumor cells. Indeed, we found that the combination of T+P in the presence of non-heat-inactivated serum significantly reduced the viability of multiple HER2^+^ cell lines in a few hours (Figure 3C). This phenomenon was also observed in murine cells expressing human HER2 (NIH-3T3-HER2 and MM3MG-HER2Δ16), suggesting that HER3 expression was not required for this activity. Furthermore, murine versions of the HER2 mAb combination (4D5-IgG2A + 2C4-IgG2A) were also capable of synergistically inducing CDC killing of HER2^+^ target cells (Supplemental Figure 6B).

**Figure 4:**
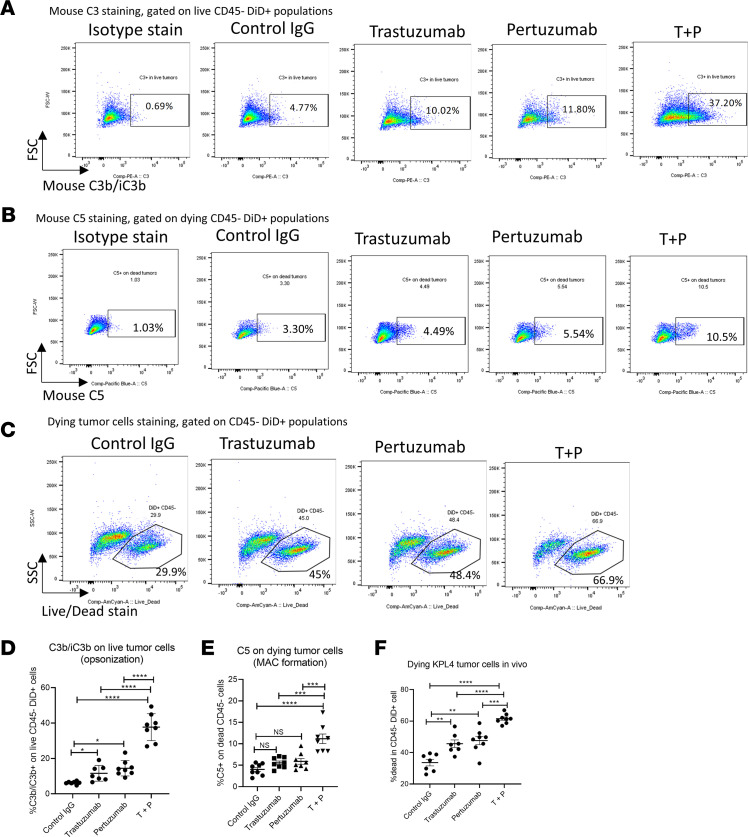
T+P combination therapy mediates complement activation, tumor cell opsonization, and complement-dependent cytotoxicity in vivo. Quantification of C3 and C5 depositions on HER2^+^ BC cells in vivo. KPL-4 cells were labeled with Vybrant DiD dye and implanted into SCID-beige mice. Once tumor volume reaches ~300 mm^3^, mice were treated with HER2 mAbs (200 μg each) or control IgG. The next day, tumors were harvested and surface stained for mouse C3 and C5, and quantified by FACS. C3^+^ staining was gated on live CD45^–^DiD^+^ cells. C5^+^ staining was gated on dying CD45^–^ cells. (**A**) Representative FACS quantification plot of C3 deposition on live KPL-4 tumor cells in vivo. (**B**) Representative FACS quantification plots of C5 staining on dying KPL-4 tumor cells in vivo. (**C**) Representative FACS quantification plots of dying KPL-4 tumor cells in vivo. (**D**) Summary of in vivo mouse C3 deposition on KPL-4 tumors. (**E**) Summary of in vivo mouse C5 deposition on KPL-4 tumors. (**F**) Summary of dying KPL-4 tumors cells frequency from above experiment. (**D**–**F**) *n* = 7–8; 1-way ANOVA with Tukey’s multiple-comparison post hoc test. All data represent mean ± SEM; *P < 0.05, **P < 0.01, ***P < 0.001, ****P < 0.0001.

To verify that this phenomenon occurs in vivo, we labeled KPL-4 cells with Vybrant DiD dye, implanted them into mouse MFPs and treated mice with HER2 mAbs once tumors were established (>200 mm^3^). Flow cytometry analysis revealed a robust increase of mouse C3b/iC3b deposition on DiD-labeled tumor cells from mice treated with T+P (Figure 4, A and D) compared with monotherapy of each mAb. Furthermore, this combination therapy also increased the MAC formation on dying tumor cell surfaces, as demonstrated by C5 staining (Figure 4, B and E). We also found an increase in human C3 and C5 depositions by T+P treatment in vivo (Supplemental Figure 6C), and this is likely from C3 and C5 production by the KPL-4 human xenograft cells, suggesting this MOA in vivo is conserved between human and mouse complement proteins. Lastly, a higher frequency of dying DiD^+^ tumor cells (but not CD45^+^ immune cells) was observed in mice receiving the combination therapy (Figure 4, C and F), indicating an increase of direct tumor cell lysis mediated by CDC. The same pattern was observed in vivo using murine implantable 3T3-HER2 tumors (Supplemental Figure 6D) and spontaneous HER2Δ16 BCs treated with murinized HER2 mAbs 4D5-IgG2A plus 2C4-IgG2A (Supplemental Figure 6E). Overall, these results reveal that activation of the complement cascade by T+P combination therapy results in tumor cell opsonization, MAC formation, and direct tumor cell lysis.

### T+P combination therapy boosts tumor phagocytosis through complement activation and tumor opsonization.

**Figure 5:**
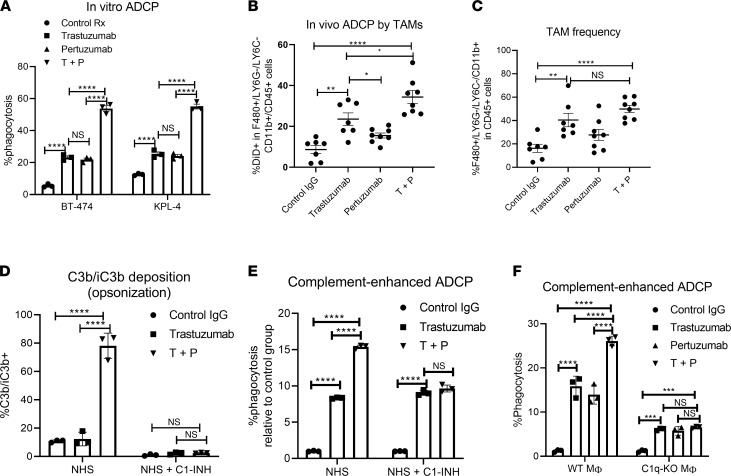
T+P combination therapy boosts tumor phagocytosis through complement activation and tumor opsonization. (**A**) HER2 mAb–induced antibody-dependent cellular phagocytosis (ADCP) of HER2^+^ BC cells by mouse BM-derived macrophages (BMDM) in vitro. KPL-4 or BT-474 cells were labeled with Brilliant Violet 450 Dye (BV450), and cocultured with BMDM with control or HER2 mAbs (10 μg/mL). ADCP rates were measured by percentage of BMDM uptake of labeled tumor cells (CD45^+^ and BV450^+^). *n* = 3. (**B**) In vivo ADCP experiment. KPL-4 cells were labeled with Vybrant DiD dye and implanted (1 × 10^6^ cells) into mammary fat pads of SCID-beige mice. Once tumor volume reached ~300 mm^3^, mice were treated with HER2 mAbs (200 μg each) or control IgG. The next day, tumors were harvested and analyzed by FACS. TAMs that had phagocytosed DiD-labeled tumor cells were quantified for ADCP. (**C**) Tumor-associated macrophages (TAMs), identified as CD11b^+^F480^+^LY6G^–^LY6C^–^, were quantified. *n* = 7–8; 1-way ANOVA with Tukey’s multiple-comparison post hoc test. (**D**) HER2 mAb–induced C3b deposition is dependent on C1q activation. KPL-4 cells were incubated with HER2 mAbs and NHS to promote C3b deposition as described in Figure 3. Where indicated, cells were treated with or without C1 inhibitor (C1-INH) to inhibit C1 activation and C3b deposition. *n* = 3. (**E**) C3b-opsonized antibody-bound KPL-4 in **D** were cocultured with human macrophages to assess complement-enhanced ADCP. *n* = 3. (**F**) KPL-4 cells were cultured with BMDMs derived from WT or C1q-deficient mice, and ADCP activity with HER2 mAbs were quantified as before. *n* = 3. (**A** and** D**–**F**) Data are shown as mean ± SEM, 2-way ANOVA with Tukey’s multiple-comparison post hoc test.

The activation of complement and opsonization of tumor cells in vivo suggested that ADCP may be similarly enhanced through complement phagocytosis. To study this possibility, we assessed ADCC and ADCP following T+P therapy. Notably, we observed a significant increase in tumor ADCP by mouse macrophages in vitro with the combination compared with single mAb treatments (Figure 5A). This phenomenon was observed using different HER2^+^ BC target cells, as well as using the murinized mAbs 4D5-IgG2A and 2C4-IgG2A (Supplemental Figure 7A). Importantly, the increase in tumor cell phagocytosis with T+P therapy was also seen using human macrophages (Supplemental Figure 7B). In addition, we found a modestly increased secretion of cytokines and chemokines (e.g., IL-6, TNF-α, MIP-1) by macrophages with the combination therapy compared with monotherapy (Supplemental Figure 8), likely a result of their enhanced ADCP of tumor cells. In contrast to ADCP, ADCC-mediated tumor lysis by NK.92 cells expressing hFCGR3A was not further increased in the T+P combination treatment (Supplemental Figure 7C). To verify the effect of ADCP in vivo, we used a previously described assay (20). In brief, we labeled KPL-4 tumor cells with Vybrant DiD dye, implanted them into the mouse MFPs, treated established tumors with HER2 mAbs, and assessed tumor phagocytosis after 24 hours by flow cytometry. We quantified ADCP by identifying DiD^+^ cells within the live macrophage populations (F4/80^+^LY6G^–^LY6C^–^CD11b^+^CD45^+^) in the tumor microenvironment. Using this technique, we found that the T+P combination therapy showed the strongest increase of tumor phagocytosis in vivo compared with either HER2 mAb alone (Figure 5B), consistent with our findings in vitro. Trastuzumab monotherapy also elicited a more significant induction of ADCP activity, in comparison with pertuzumab monotherapy in vivo (Figure 5B), consistent with its superior activation of mouse FCGR4 (Figure 2C). Tumor-associated macrophage (TAM) levels were also elevated with the combination therapy compared with single mAb treatment, although the increase of TAM levels observed did not achieve statistical significance (Figure 5C).

Since we did not observe increased activation of FCGRs with T+P treatment (Figure 2, A–G) but did observe activation of the classical complement pathway (Figures 3 and 4), we hypothesized that complement-mediated phagocytosis could be complementing ADCP. To determine the role of complement enhanced phagocytosis, we utilized a C1-inhibitor (C1-INH) that blocks activated C1 through sequestering and removing C1r and C1s from the C1 complex (38). We first confirmed that the addition of C1-INH in NHS prevented KPL-4 cells from becoming opsonized by T+P therapy (Figure 5D). As hypothesized, the increased tumor phagocytosis with the combined HER2 mAbs treatment was abolished in the presence of C1-INH (Figure 5E). Macrophages were most likely the source for complement proteins in our ADCP assays, as they are known to secrete high levels of classical complement proteins after FCGR activation (39). To further confirm the role of classical complement pathway in enhancing tumor phagocytosis, we assessed complement enhanced ADCP using BM-derived macrophages (BMDM) generated from C1q-deficient (C1q-KO) mice. Notably, the boost in tumor phagocytosis following T+P treatment was completely abolished with C1q-KO macrophages, indicating the requirement of classical complement pathway activation for the complement-enhanced phagocytosis seen in T+P therapy (Figure 5F). These experiments indicate that the activation of the complement system and tumor opsonization by T+P combination therapy could further enhance tumor phagocytosis by macrophages, in addition to their direct engagement with FCGR to trigger ADCP.

### The significant role of classical complement pathway activation in T+P therapeutic efficacy in vivo.

**Figure 6:**
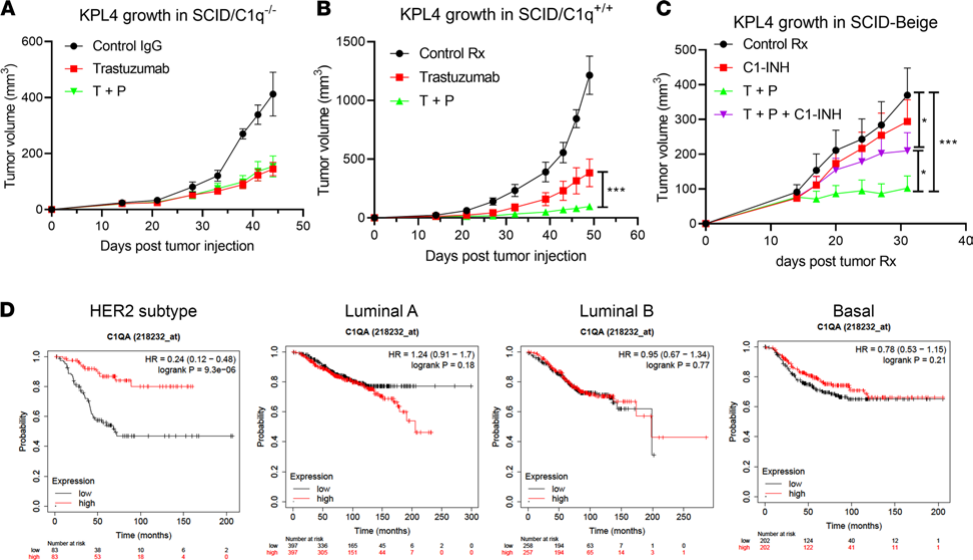
The significant role of classical complement pathway activation in T+P therapeutic efficacy in vivo. (**A**) Immunodeficient C1q-KO mice (SCID/C1q^–/–^) were implanted with KPL-4 cells in the mammary fat pads (5 × 10^5^ cells). HER2 mAbs (100 μg each) or control human IgG1 was administered weekly, and tumor volume was measured. Data are shown as mean ± SEM. Control IgG group, *n* = 5; Trastuzumab group, *n* = 14; T+P group, *n* = 14. (**B**) Same experiment as in **A** was repeated in SCID/C1q^+/+^ mice. Data are shown as mean ± SEM. Control IgG group, *n* = 5; Trastuzumab group, *n* = 14; T+P group, *n* = 14. (**C**) KPL-4 cells were implanted into mammary fat pads of SCID-beige mice as before (1 × 10^6^ cells). When average tumor volume reached ~100 mm^3^, mice were divided into 4 treatment groups: Control IgG, T+P (100 μg each, weekly), C1-INH (200 μg, twice per week), or T+P and C1-INH. Data are shown as mean ± SEM; *n* = 10 for all groups. (**A**–**C**) Two-way ANOVA with Tukey’s multiple-comparison post hoc test. All data represent mean ± SEM; *P < 0.05, ***P < 0.001. (**D**) The prognostic value of *C1QA* expression in BC patients. BC patients in KM plotter database were classified into HER2^+^, Luminal A, Luminal B, and Basal subtypes using St. Gallen classification. For each subtype, patients were split into 2 groups by their expression levels of *C1QA*, and the overall survival (OS) was compared between patients with high and low *C1QA* gene expression.

Having demonstrated a role for complement in mediating tumor cytotoxicity and phagocytosis, we next wanted to demonstrate the importance of classical complement pathway in HER2 mAb therapy efficacy in vivo. As C1q is the initiator of classical complement pathway activation by mAbs, we generated C1q-deficient mice (on a SCID background) to first assess T+P therapeutic synergy on KPL-4 tumor growth in the MFP. As expected, we found no difference between the T+P combination and monotherapy antitumor efficacy in C1q-deficient mice (Figure 6A), suggesting the synergistic antitumor effects of T+P therapy requires C1q. In parallel crossbreeding, we generated control C1q^+/+^ mice on a SCID background and showed that T+P therapy retains its enhanced antitumor efficacy over monotherapy in C1q-intact mice (Figure 6B). Flow cytometry analysis confirmed the lack of tumor opsonization by C3b and direct tumor cell death in C1q-deficient mice but not C1q^+/+^ mice after T+P treatment (Supplemental Figure 9). To further confirm the role of classical complement pathway activation in T+P antitumor activity, we treated tumor-bearing mice with the C1-INH described earlier (38). While C1-INH itself has no effect on KPL-4 tumor growth, we found that C1-INH treatment significantly reduced T+P therapeutic activity in vivo (Figure 6C). Collectively, these data illustrate that the activation of C1 and the downstream classical complement cascade are crucial components for the antitumor MOA of T+P combination therapy.

Based on these findings, we hypothesized that expression of C1q genes (i.e., *C1QA*, *C1QB*, and *C1QC*) may be associated with the efficacy of HER2^+^ subtype BC patients receiving HER2-targeted therapy. To investigate this hypothesis, we utilized 2 survival analysis databases for BC—KMplot (40) and BreastMark (41)—and assessed the relationship between C1q expression and overall survival (OS) outcomes for patients with HER2^+^, Luminal A, Luminal B, and Basal (triple-negative BC [TNBC]) subtypes. These analyses reveal that the expression of multiple C1q genes (i.e., *C1QA*, *C1QB*, and *C1QC*) associate with a better OS outcome in patients with HER2^+^ BC, compared with TNBC or Luminal A/B subtypes (Figure 6D and Supplemental Figures 10 and 11). This supports a possible role for classical complement genes in positive survival outcomes and indicates that they may serve as important biomarkers in future studies.

### Inhibition of complement-regulatory proteins CD55 and CD59 sensitizes HER2^+^ BC to T+P combination therapy.

**Figure 7:**
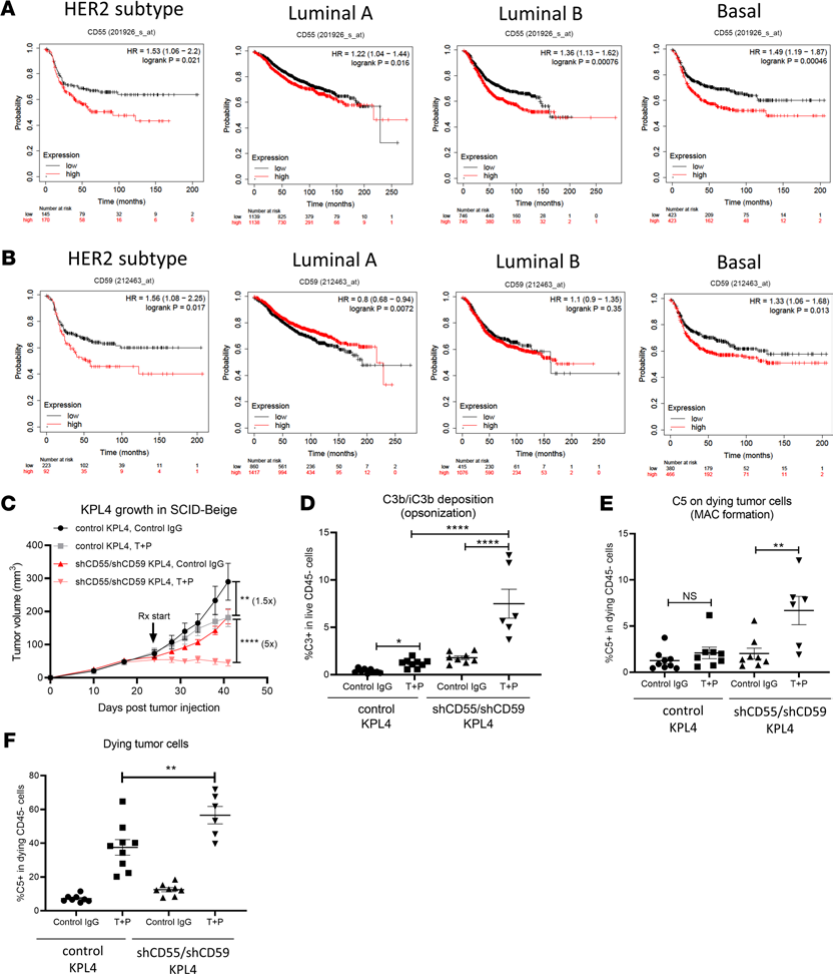
Inhibition of complement-regulatory proteins CD55 and CD59 sensitizes HER2^+^ BC to T+P combination therapy. (**A** and **B**) The prognostic value of *CD55* and *CD59* expression in breast cancer patients. BC patients in KM plotter database were classified into HER2^+^, Luminal A, Luminal B, and Basal subtypes using St. Gallen classification. For each subtype, patients were split into 2 groups by their expression levels of *CD55* or *CD59*, and the relapse-free survival (RFS) was compared between patients with high and low gene expression. (**C**) Control KPL-4 or CD55/CD59 double-knockdown KPL-4 cells were implanted into mammary fat pads of SCID-beige mice (5 × 10^5^ cells). When average tumor volume reached ~100 mm^3^, mice were divided into 2 treatment groups: Control IgG and T+P (25 μg each, weekly). Data are shown as mean ± SEM; *n* = 9 for all groups. Two-way ANOVA with Tukey’s multiple-comparison post hoc test. (**D**–**F**) In vivo complement deposition and killing of control and knockdown KPL-4 tumors from **C**. Data are shown as mean ± SEM; *n* = 6–9 for all groups. One-way ANOVA with Tukey’s multiple-comparison post hoc test. (**D**) Summary of in vivo C3 deposition. (**E**) Summary of in vivo C5 deposition. (**F**) Summary of dying KPL-4 tumor cell frequency. All data are shown as mean ± SEM; **P < 0.01, ****P < 0.0001.

Membrane-bound CRPs such as CD55 (DAF) and CD59 (MSK21) are known to inhibit complement activity and are often upregulated in cancer (29, 30), with some prognostic capacity in HER2^+^ BC patients (42–44). Indeed, analysis using the KMplot database found that expression of both CD55 (Figure 7A) and CD59 (Figure 7B) are inversely correlated with survival outcome in BC, particularly HER2^+^ subtype BCs. Similar results were observed in the BreastMark database (Supplemental Figure 12). To investigate the effect that CD55 and CD59 may have on T+P therapeutic activity in our models, we generated and confirmed double knockdown of these genes in KPL-4 cells using stable lentiviral transduction of shRNAs (Supplemental Figure 13). Since our prior T+P treatment of KPL-4 xenografts resulted in near-complete tumor regression, we reduced and delayed our antibody therapy regimen for this experiment. We found that, while this reduced dose of T+P could only moderately inhibit control KPL-4 tumor growth, knockdown of CD55 and CD59 in the same tumors resulted in profound tumor growth suppression (Figure 7C). Analysis of complement activity within treated tumors revealed that CD55/CD59 double-knockdown resulted in a further increase of C3b deposition (Figure 7D), MAC formation (Figure 7E), and direct tumor lysis after T+P therapy (Figure 7F). This experiment demonstrates that local knockdown of CD55/CD59 can improve complement-mediated antitumor activity of T+P therapy. Our data suggest the clinical relevance of CRPs in T+P therapy outcomes and support the potential use of targeted CRP inhibition to improve antitumor mAb combinations.

## Discussion

Phase III clinical studies demonstrate the superior outcomes by adding pertuzumab to a trastuzumab plus chemotherapy regimen in both metastatic and early-stage HER2^+^ BC (8, 9), leading to the US FDA approval of pertuzumab in conjunction with trastuzumab and chemotherapy in 2012. While this combination therapy remains the standard of care for most newly diagnosed HER2^+^ BC, the MOA responsible for the combination has been the subject of speculation (45) but remains poorly understood. While initial studies demonstrate the ability of pertuzumab to selectively inhibit HER2 heterodimerization with different ErbB receptors (12–15), investigations demonstrating the MOA responsible for the in vivo therapeutic effect of pertuzumab have been lacking. Similar to trastuzumab, pertuzumab is a humanized IgG1 mAb and is theoretically capable of strongly activating Fc-mediated effector functions such as ADCC, ADCP, and CDC, as demonstrated in previous studies (16, 37). However, none of these studies have linked these immunologic mechanisms to the therapeutic efficacy of pertuzumab. Additionally, while both preclinical and clinical studies have demonstrated synergies between trastuzumab and pertuzumab (5, 6, 8, 16), which are often ascribed to their potentially different individual MOAs (13, 17), there is a lack of studies investigating potential unique synergies or emergent MOA from the combined use of these antibodies. Given our recent findings involving the ADCP MOA for trastuzumab (20), we utilized our potentially novel models and approaches to interrogate the in vivo antitumor mechanism of pertuzumab in combination with trastuzumab against HER2-driven BC.

In this study, we used multiple models of human HER2–expressing tumors—i.e., KPL-4, NIH/3T3-HER2, SKOV3, MM3MG-HER2Δ16, BT-474, and an endogenous transgenic HER2+ BC model that is tolerant to human HER2. In these models, we tested both clinical-grade pertuzumab and the murine version of pertuzumab with the functionally equivalent mouse isotype (2C4-IgG2A) to demonstrate the different MOA of pertuzumab in treating HER2^+^ BC. We first confirm that pertuzumab inhibits HER2/HER3 heterodimerization-mediated oncogenic signaling (Supplemental Figure 4), as reported before (12, 14, 15). We also demonstrate that pertuzumab as a single agent can activate both ADCC and ADCP to elicit antitumor activity, though with weaker FCGR activation and in vivo ADCP efficacy compared with trastuzumab (Figure 2, A–G; Figure 5, A and B; and Supplemental Figure 7). In contrast, our studies of the T+P combination therapy demonstrate the synergistic activation of classical complement pathways, allowing for direct lysis (CDC) of HER2^+^ tumor cells and enhanced tumor phagocytosis (CDCP) by macrophages.

The importance of complement activation for the therapeutic activity of T+P therapy in vivo is supported by the following findings: (a) the synergistic therapeutic effect of pertuzumab in combination with trastuzumab occurs in HER2^+^ BC without expression of other ErbB receptors and does not require the presence of adaptive immunity or NK cells (as shown using SCID-beige mice); (b) complement activation requires both trastuzumab and pertuzumab in vitro and is eliminated through the use of C1-INH or serum heat-inactivation, while in vivo T+P synergistic efficacy requires intact C1q and the intact Fc domains of both mAbs; (c) T+P therapy elicits robust complement deposition on tumor cells, MAC assembly, and direct tumor cell lysis, as well as complement-enhanced phagocytosis in vitro and in vivo; (d) C1q gene expression associates with favorable survival outcome in HER2^+^ subtype BC patients, whereas inhibition of C1 activity in mice reduced T+P antitumor activity; and (e) suppression of complement regulatory genes (CD55, CD59) on tumor cells sensitizes HER2^+^ tumors to T+P therapies, whereas CD55/CD59 expression negatively correlates with survival in HER2^+^ BC, although not in other molecular subtypes.

Congruent with our findings, complement activation has been demonstrated by other approved therapeutic antibodies (such as Rituximab and Cetuximab) and has been suggested to contribute to their antitumor therapeutic efficacies (46–49). In addition, elevated expression of complement regulators has been noted on many cancers (50, 51), with negative associations noted in BC and specifically in HER2^+^ BC (42–44), in agreement with our own analysis.

Our study highlights a previously underappreciated complement-dependent MOA for T+P therapies that may be critical for the clinical efficacy of the standard-of-care treatment of most HER2^+^ BC. Moreover, it suggests that strategies to exploit complement activation might find utility in HER2^+^ BC and other solid cancers that do not currently have developed mAb therapies. While complement was originally recognized as an innate immune humoral defense system that aided or “complemented” antibodies, recent studies have demonstrated how tumors can co-opt inflammatory factors elicited by complement to aid their growth (50, 52–54). Indeed, recent studies have demonstrated that the opposing roles of complement in tumors are likely determined by the specifics of the tissue type, specific type of activation, and overall composition of the tumor microenvironment (50, 51, 53). As such, the efficacy of therapeutic strategies that function through complement will likely vary between different cancers. Interestingly, exploration of gene expression data has revealed evidence that most tumors have elevated expression of complement regulators, such as CD55 and CD59, while also having low expression of complement genes (50, 51). This suggests a concerted modulation of complement activity that is conserved in most cancers. The differential expression of complement genes and regulators could explain why some types of HER2^+^ tumors are more resistant to T+P therapies. For instance, negative regulators of complement (such as CD46, CD55, and CD59) have been found to be highly expressed in gastric cancers (55–57), which could potentially explain why T+P therapies failed to offer a significant clinical benefit in the JACOB Phase III trials of gastric HER2^+^ cancers (58). While speculative, the demonstrated clinical utility of T+P therapies in BC, along with the development of targeting strategies to locally inhibit CRPs, could offer a path forward to enhance combination mAb therapy against other previously resistant types of cancers.

Our study also demonstrates that 2 IgG1 isotype mAbs for an identical target (HER2) have different capacities to inhibit HER2 signaling and activate FCGRs, while requiring their combination to activate the classical complement cascade. The variability in activity is likely due to differences in affinity and binding orientations. Of note, high-resolution crystallography has demonstrated how IgGs form hexameric structures by interacting with neighboring IgG molecules, which leads to recruitment and activation of C1, thereby triggering the complement cascade (59). The close proximity of trastuzumab and pertuzumab when both are bound to highly expressed HER2 proteins on the tumor cell surface appears capable of triggering this interaction, which may have implications for the future development of mAb combinations.

In addition to direct tumor lysis, the activation of the classical complement pathway also induces diverse immune effector functions, including phagocytosis and anaphylatoxins release (22–25). In particular, C3b/iC3b deposition on tumor surfaces serves as an important opsonin to enhance phagocytosis upon engaging with complement receptors on phagocytes (29). The downstream soluble product C5a, upon binding to C5aR, could also enhance mAb-mediated effector functions such as ADCP and macrophage activation, as suggested by other studies (60, 61) and confirmed by our studies (Figure 5). While these anaphylotoxins and their receptors can also impact adaptive immunity such as DC, B cell, and T cell function (24, 25, 62), our studies of immunocompetent HER2 transgenic mice did not reveal drastic changes in lymphocyte infiltration (Supplemental Figure 14) or HER2-specific T cell adaptive responses with T+P therapy, despite an increase in complement activation, tumor cell death, and improved survival in these mice (Figure 1E and Supplemental Figure 6E). Nevertheless, clinical studies have demonstrated significant associations between HER2-specific adaptive immune responses and HER2-targeted therapy plus chemotherapy (63). Therefore, further investigations are required to determine whether T+P combination therapy could influence adaptive immune responses against HER2^+^ BC, especially in the context with immunogenic chemotherapy.

Our study highlights that T+P therapies offer multiple individual MOA, including eliciting HER2 signaling blockade, ADCC, and ADCP, in addition to the emergent properties seen with T+P combination in stimulating CDC and CDCP to collectively elicit potent antitumor effects. While the clinical success of T+P therapy for HER2^+^ BC offers a potential path forward in treating other solid cancers, it suggests that combinations may require all these activities to achieve comparable clinical success. These insights may allow for additional approaches to enhance complement-mediated antitumor immunity through targeted blockade of complement regulators (64–66) or through Fc-engineering to enhance C1q binding and activation (67). However, complement activation can be a double-edged sword and may cause unwanted toxicity and complications. For example, for patients undergoing breast reconstruction surgeries after treatment, this combination (T+P) has been independently associated with higher risk of postoperative wound breakdown requiring additional surgical intervention (68). This may be attributable to the activation of complement and local inflammation, which has been demonstrated to suppress wound healing (69, 70). This may be an important consideration in formulating temporal guidelines for patients undergoing breast reconstruction after T+P therapy. Indeed, a systematic review and metaanalysis revealed that T+P combination with or without chemotherapy is associated with a higher risk of toxicities compared with trastuzumab with or without chemotherapy; toxicities include rash, diarrhea, mucosal inflammation, and anemia (71)—all symptoms that are consistent with complement-mediated inflammation and tissue damage (72–74). Fortunately, there are no widespread reports of systemic complement activation or thrombosis with this combination. This may be due to the requirement for a threshold level of antibody complexes to be formed, which may be absent in nontumor tissue expressing low levels of HER2.

In summary, our study demonstrates an emergent MOA for the T+P combination therapy in their combined ability to activate the classical complement pathway that does not occur with either antibody used in isolation. Specifically, increased complement deposition and tumor opsonization after T+P therapy can enhance tumor phagocytosis and stimulate direct tumor cell lysis. These findings have potential implications for the use of T+P in HER2^+^ cancers, where modulating complement activity may improve efficacy and monitoring complement-related genes may provide a biomarker to guide clinicians. These results also raise the bar for the implementation of effective monoclonal antibody combinations in other cancers, where the role of complement activity and regulation is not well understood.

## Methods

Supplemental Methods are available online with this article.

### Cell lines.

Human HER2^+^ BC cell line KPL-4 was a gift from Junichi Kurebayashi (University of Kawasaki Medical School, Kurashiki, Japan) (75). Human HER2^+^ BC cell lines (BT-474, SKBR3, and Au565) and HER2^+^ ovarian cancer cell line SKOV3 were obtained from ATCC and cultured as described by their ATCC protocol. The mouse fibroblast NIH/3T3 cell line stably expressing human HER2 was generated using lentiviral transduction (pCDH-CMV vector) and cultured in DMEM +10% bovine calf serum. NIH/3T3-HER2 cells were further purified by FACS, and > 80% HER2^+^ frequency was obtained after FACS (Supplemental Figure 2A) and used for in vivo experiments. The Jurkat-NFAT-Luciferase line for FCGR activation assays were obtained from Invivogen (jktl-nfat).

### In vitro cell growth inhibition.

Various HER2^+^ tumor model cell lines (KPL-4, AU565, SKOV3, BT-474, 3T3-HER2) were plated at 10%–30% confluency, treated with 20 μg/mL of HER2 mAbs (trastuzumab, pertuzumab, or combination), and cultured for 6 days. Fresh media with HER2 mAbs were changed every 2 days. Cell viability was analyzed by CellTiter-Glo assay on day 6.

### HER2 signaling assay.

HEK 293T cells stably expressing HER2 and HER3 were transfected (Lipofectamine 3000) with luciferase reporter constructs (5 μg of DNA in 2 × 10^6^ cells) for MAPK/ERK or AP-1/c-JUN pathway activation. Reporter constructs were originated from Cignal Reporter Assay Kit (336841, Qiagen). After 12 hours of transfection, cells were treated with 50 ng/mL Heregulinβ-1 (HRG1-β1), and HER2 mAbs or lapatinib (kinase inhibitor of HER2 signaling as assay positive control) at the concentrations as indicated in the figures. HER2 signaling activity was analyzed by luciferase readout of MAPK/ERK and AP-1/c-JUN pathway reporters. Cells without HRG1-β1 treatment were used as negative controls.

### FCGR binding/activation assay.

Jurkat cells expressing FCGRs (mouse *FCGR1*, *FCGR3,* and *FCGR4* or human *FCGR1* and *FCGR3A-V158*) with NFAT-Luciferase reporter were generated with lentiviral transduction and selected with puromycin. To assess FCGR activation, KPL-4 BC lines were first plated and treated with 10 μg/mL of control IgG or HER2 mAbs for 1 hour. Jurkat-FCGR-NFAT-Luciferase effector cells were added and cocultured for 4 hours. FCGR signaling activation was assessed by luciferase activity quantification.

### In vitro complement activation and deposition analysis.

HER2^+^ cell lines (KPL-4, BT-474, and SKBR3) were incubated with 10 μg/mL HER2 mAbs for 1 hour at 37°C. After incubation, NHS (CompTech) was added to culture to a final concentration of 10% serum. When indicated, C1-INH (CompTech, A140) was added to culture at 20 μg/mL. C1q-depleted serum was purchased from CompTech (A300). Cells were cultured at 37°C for another 30 minutes, washed once with PBS, and stained with anti–human C3b FITC (BioLegend, 846107) and viability dye (Aqua) for FACS analysis of live C3b^+^ cells percentage.

### In vitro CDC assay.

HER2^+^ target cells were incubated with 10 μg/mL HER2 mAbs for 1 hour at 37°C. After incubation, human or rabbit serum (non-heat-inactivated) were added to culture to a final concentration of 25% serum. After 4–6 hours, cell viability was assessed using a CellTiter-Glo luminescent assay. Heat-inactivated serum was used as negative control.

### In vitro ADCP assay.

ADCP assays by macrophages were performed as described before (20). Briefly, human monocyte-derived macrophages (MDM) were generated from donor PBMCs. MDM were generated with 50 ng/mL human M-CSF (Peprotech, 300-25) and 50 ng/mL human GM-CSF (Peprotech, 300-03). When mouse macrophages were used, cells were isolated from mouse BM and differentiated with 50 ng/mL murine M-CSF (Peprotech, 315-02) to generate BMDM. HER2^+^ tumor cells were labeled with Brilliant Violet 450 Dye (BD Biosciences, 562158), according to manufacturer protocol, and incubated with control or HER2 mAbs (10 μg/mL) in 96-well plates (100,000 cells/well) for 30 minutes at 37°C. When indicated, C1-INH were added at 20 μg/mL. Macrophages were then added for coculture at a 1:2 ratio of tumor/macrophages. After 2 hours of coculture, phagocytosis of Brilliant Violet 450 Dye–labeled (BV450-labeled) tumor cells by MDM were analyzed by FACS with CD45-APC or CD11b-PECy7 staining and LIVE/DEAD Fixable Red. 

### In vitro ADCC assay.

CEM.NKR cells stably expressing HER2 and luciferase (CEM.NKR-HER2), and NK.92 cells stably expression human FCGR3 of the V158 variant (NK.92-FCGR3-V158) were generated with lentivirus transduction and selected with puromycin. For the ADCC assay, CEM.NKR-HER2 (100,000 cells/well) were incubated with 10 μg/mL HER2 mAbs for 30 minutes. NK.92-FCGR3-V158 (10,000 cells/well) were then added for coculture. Target cell viability after 24-hour coculture was analyzed by luciferase activity measurements. When indicated, CEM.NKR not expressing HER2 were used as negative controls.

### Mice.

SCID-beige (C.B-*Igh*-1b/GbmsTac-*Prkdc^scid^-Lyst^bg^* N7) were purchased from Taconic Biosciences (model CBSCBG). C1qa-KO mice (B6[Cg]-C1qa^tm1d(EUCOMM)Wtsi^/TennJ) were purchased from The Jackson Laboratory (stock no. 031675). C1qa-KO mice were crossed with Balb/c SCID mice to obtain SCID/C1qa^–/– ^and SCID/C1qa^+/+^ strains. All mice used for experiments were females between the ages of 6 and 12 weeks old. The HER2Δ16 transgenic model was generated by crossing MMTV-rtTA strain (in-house) with TetO-HER2d16-IRES-EGFP strain (in-house) (33).

### Therapeutic antibodies.

Clinical grade T+P was obtained from Duke Medical Center. The murine versions of trastuzumab (4D5) and pertuzumab (2C4) with the IgG2A mouse isotypes were produced by GenScript through special request. F(ab’)_2_ of T+P antibodies were generated by Pepsin digestion using the Pierce F(ab’)_2_ Preparation Kit (Thermo Fisher Scientific, 44988). Manufacturer protocol was followed for both digestion and purification.

### Orthotopic implanted HER2^+^ BC mouse models and therapeutic antibody treatments.

All HER2^+^ cell lines were suspended in PBS + 50% matrigel (Corning, 354234), and 50 μL total volume was used per injection into immunodeficient mouse models. KPL-4 cells (5 × 10^5^ cells) were implanted into MFP of female mice. NIH/3T3-HER2 (2 × 10^5^ cells) and SKOV3 (1 × 10^6^ cells) were injected s.c. in the flanks of mice. Tumor growth was measured with caliper-based tumor volume measurement (length × width × width/2) over time. For therapeutic treatments, T+P and their F(ab’)_2_ counterparts were administered weekly (100 μg of each antibody per mice i.p.) when average tumor volume reached between 30 and 80 mm^3^, unless otherwise indicated. C1-INH treatments were used at 200 μg per mice i.p., twice per week. For experiment using CD55/CD59 double-knockdown KPL-4 tumors, 25 μg of each antibody (T+P) was administered weekly, and treatment was started when average tumor volume reached about 100 mm^3^.

### Transgenic HER2Δ16 mouse model and therapeutic antibody treatments.

The HER2Δ16 transgenic mouse model was generated by crossing 2 strains of mice, TetO-HER2Δ16-IRES-EGFP and MMTV-rtTA. This system was described previously (33) but utilizes a TET-ON system (with MTV-rtTA) to drive expression of HER2Δ16 to generate HER2^+^ BC. For experiments, 1-month-old mice were put on doxycycline diet (200 mg/kg, Bio-Serv) to induce spontaneous HER2-driven BC. Individual animals were randomly enrolled into a specific treatment group as soon as palpable breast tumors were detected (~100 mm^3^) in any of the 8 MFP. Control IgG, 4D5-IgG2A, and 2C4-IgG2A antibodies were treated weekly (200 μg per mice for each antibody). Animals were terminated once their total tumor volume reached > 2000 mm^3^.

### Flow cytometry analysis of tumor infiltrating immune cells.

When tumor growth reached humane end point size (>500 mm^3^), whole tumors from mice were harvested and cut into < 1 mm pieces and incubated for 1 hour in digestion buffer (DMEM + 100 μg/mL collagenase + 0.2 U/mL DNAse + 1 μg/mL hyalurodinase). Single-cell suspensions were spun down through a 70 μm filter and washed with medium. Approximately 1 million to 5 million cells were used for staining and flow cytometry analysis. The following panel of immune cell markers (BioLegend) were used: CD45 BV605, CD11b PE-Cy7, LY6G APC, LY6C BV410, F4/80 PerCP-CY5.5, CD4 PE-TR, CD8 APC-Cy7, and viability dye (Aqua). Cells were fixed in 1% paraformaldehyde and analyzed on the LSRII flow cytometry machine. TAM were identified by F4/80^+^LY6G^–^LY6C^–^CD11b^+^CD45^+^ live gating. Tumor-infiltrating CD8 T cells were identified as live CD45^+^CD11b^–^CD8^+^CD4^–^. CD4 T cells were identified as live CD45^+^CD11b^–^CD8^–^CD4^+^, and NK cells were identified as live CD45^+^CD11b^–^CD8^–^CD4^–^CD49b^+^.

### In vivo ADCP assay.

KPL-4 cells were labeled with Vybrant DiD labeling solution (Thermo Fisher Scientific, V22887) according to manufacturer protocol, and labeled cells were implanted (1 × 10^6^) into MFP of SCID-beige mice. Once a tumor reached around 500 mm^3^, mice were treated with HER2 mAbs (200 μg each) or with control human IgG1. TAMs were analyzed by FACS (live CD45^+^, CD11b^+^, F4/80^+^, LY6G^–^, LY6C^–^) as described above, and the percentage of TAMs that have taken up DiD-labeled tumor cells was quantified for in vivo ADCP analysis.

### In vivo complement activation analysis.

KPL-4 cells were labeled with Vybrant DiD labeling solution (Thermo Fisher Scientific, V22887) according to manufacturer’s protocol, and labeled cells were implanted (1 × 10^6^ cells) into MFP of SCID-beige mice. Similarly, NIH-3T3-HER2 cells were labeled with Vybrant DiD and implanted (2 × 10^5^ cells) into the flank of SCID-beige mice. Once a tumor reached around 500 mm^3^, mice were treated with HER2 mAbs (200 μg each) or with control human IgG1. For in vivo mouse complement deposition staining, tumor cell suspensions were prepared as described above and stained with 2 μg/mL of rat anti–mouse C3 antibody (clone 11H9, Abcam, ab11862) and 2 μg/mL rabbit anti–mouse C5 antibody (Abcam, ab11898) in 1% BSA/PBS at 4°C for 30 minutes. Cells were washed with PBS and stained with secondary antibodies goat anti–rat-PE (BioLegend 405406) and goat anti–rabbit-BV421 (BioLegend 406410) at 1:1000 dilutions for 30 minutes at room temperature (this panel also contains staining for viability dye [Aqua] and CD45-BV605 during primary antibody incubation). For in vivo human complement deposition staining, a similar procedure was followed, and the cells were stained with anti–human C3b FITC (BioLegend, 846107) and mouse anti–human C5b-9 antibody (Thermo Fisher Scientific, clone aE11). Secondary antibody goat anti–mouse-APC (BioLegend 405308) was used. Cells were also preincubated with 10 μg/mL mouse Fc-blockers (BioLegend, 101319) on ice for 10 minutes before antibody staining.

### shRNA transduction with lentivirus.

Human HER2^+^ BC cell line KPL-4 were transduced with lentivirus vector (pLKO.1) expressing shRNA against CD55 (clone TRCN0000057163, target sequence 5′-CCACACCAAATGCTCAAGCAA-3′) and CD59 (clone TRCN0000057108, target sequence 5′-CCGTCAATTGTTCATCTGATT-3′). An empty vector transduction was used as control. Transduced cells were selected with puromycin (5 μg/mL), and knockdown of CD55 and CD59 was confirmed by flow cytometry analysis using CD55-APC and CD59-PE (both from BioLegend).

### Kaplan-Meier plotter.

The relationship between complement-related gene expression and prognosis of different molecular subtypes of BC patients was analyzed using Kaplan-Meier plotter (Km plotter for BC, mRNA gene chip) (40). The “auto select best cutoff” option was used to split patients’ expression levels. The 2 groups were compared in order to determine differences in OS or relapse-free survival (RFS). BC subtypes for HER2^+^ (*n* = 515), Luminal A (*n* = 3511), Luminal B (*n* = 2015), and Basal (*n* = 1494) were determined using St. Gallen classification. The Affymetrix IDs for the following genes were selected: C1QA (218232_at), C1QB (202953_at), C1QC (225353_at), CD55 (201926_s_at), and CD59 (212463_at).

### Statistics.

All statistical analyses of tumor growth comparisons and tumor immune infiltrates were performed with GraphPad Prism (v8) using 2-way ANOVA or 1-way ANOVA with Tukey’s multiple-comparison post hoc test. Unless otherwise indicated in the figure, tests results were shown between treatment and control groups. Group sizes for animal tumor growth experiments were determined based on preliminary datasets. All subjects in animal experiments were randomized into a treatment or control group. For in vitro experiments (i.e., ADCP/ADCC/CDC assays and FCGR signaling assays), all data were statistically analyzed by 1-way ANOVA with Tukey’s multiple-comparison post hoc test, and tests were performed with at least 3 biological replicates per experiment and repeated at least 2 times. A 95% CI was considered for statistics, and *P* < 0.05 was considered significant.

### Study approval.

All animals were maintained; bred, in accordance with Duke IACUC–approved protocol (A080-20-04); and supervised by Division of Laboratory Animal Resources (DLAR).

## Author contributions

LCT, HKL, and ZCH conceived the project and wrote the manuscript. LCT designed the experiments and analyzed and interpreted the data. LCT performed the animal experiments, with assistance from EJC, XY, and CAR. TW provided technical support and suggestions for flow cytometry. ZCH generated various cell lines and CRISPR KOs, with assistance from JW and GL. The MMTV-rtTA mouse strain was provided by LAC, and TetO-HER2d16-IRES-EGFP mouse was provided by WJM. EJC and TNT provided comments, suggestions, and improvements for the manuscript. ANS provided technical support for Western blots.

## Supplementary Material

Supplemental data

## Version 1 02/15/2022

In-Press Preview

## Version 2 03/22/2022

Electronic publication
