# Supplementary material for: Trastuzumab/pertuzumab combination therapy stimulates antitumor responses through complement-dependent cytotoxicity and phagocytosis
Source: JCI Insight. 2022 Mar 22;7(6):e155636. doi: 10.1172/jci.insight.155636 (PMC8986081; doi:10.1172/jci.insight.155636)
Supplement: Supplemental data [file jciinsight-7-155636-s124.pdf]

Supplementary Figure 1

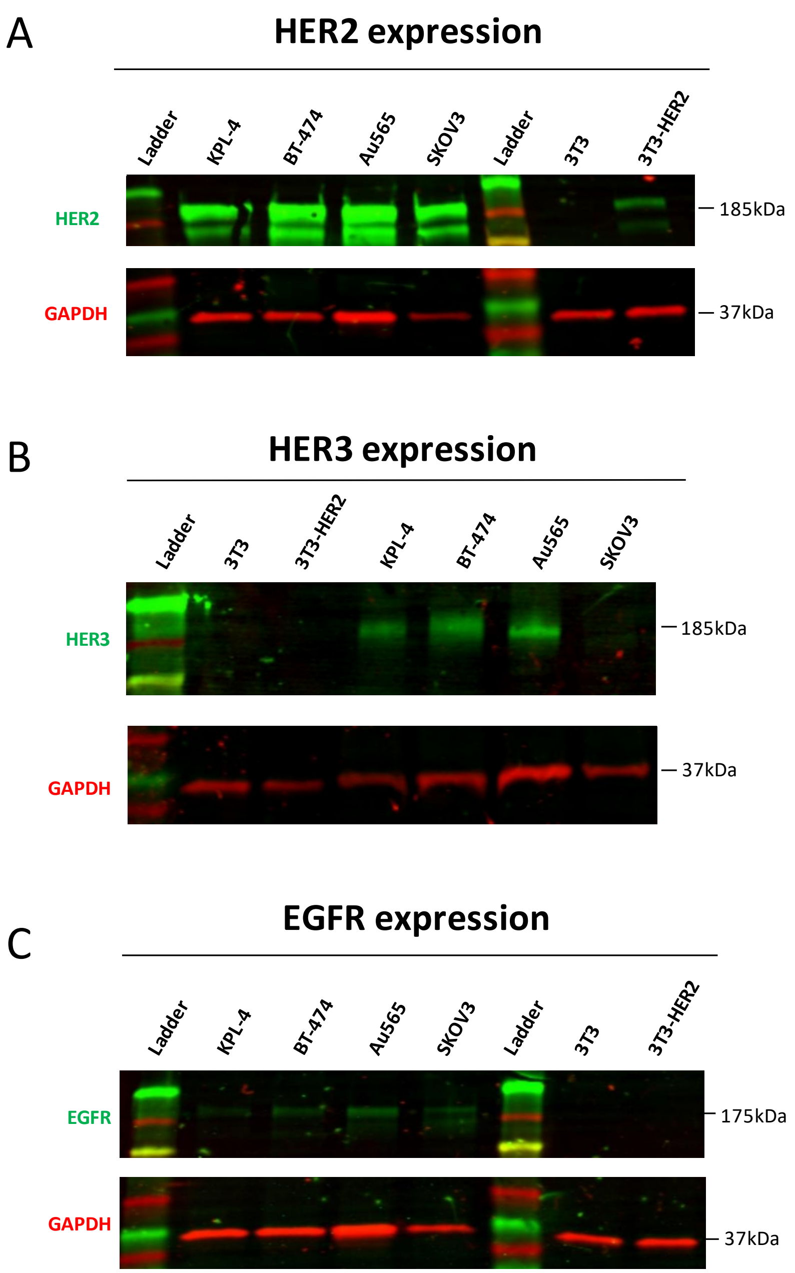

Figure S1
Western blot analysis of HER2 (A), HER3 (B) and EGFR (C) expression in KPL-4, BT-474, Au565, SKOV3, NIH/3T3 and NIH/3T3-HER2 cell lines. Blots were probed and analyzed using the LI-COR technology.

Supplementary Figure 2

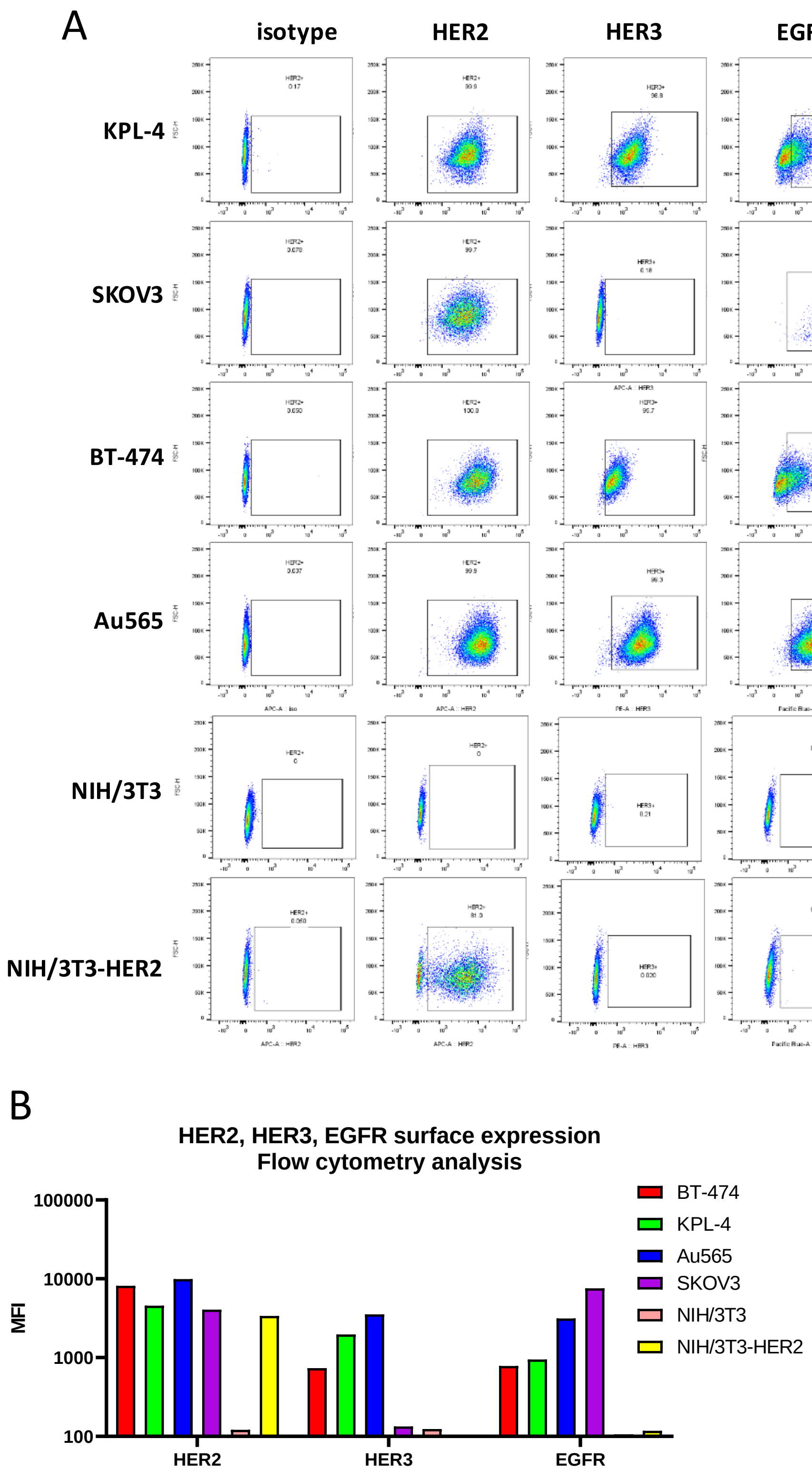

Figure S2
Flow cytometry quantification of HER2, HER3 and EGFR expression on cell surface in KPL-4, BT-474, Au565, SKOV3, NIH/3T3 and NIH/3T3-HER2 cell lines. (A) Representative flow diagrams. (B) Summary of mean fluorescent intensity (MFI) of flow staining results.

Supplementary Figure 3

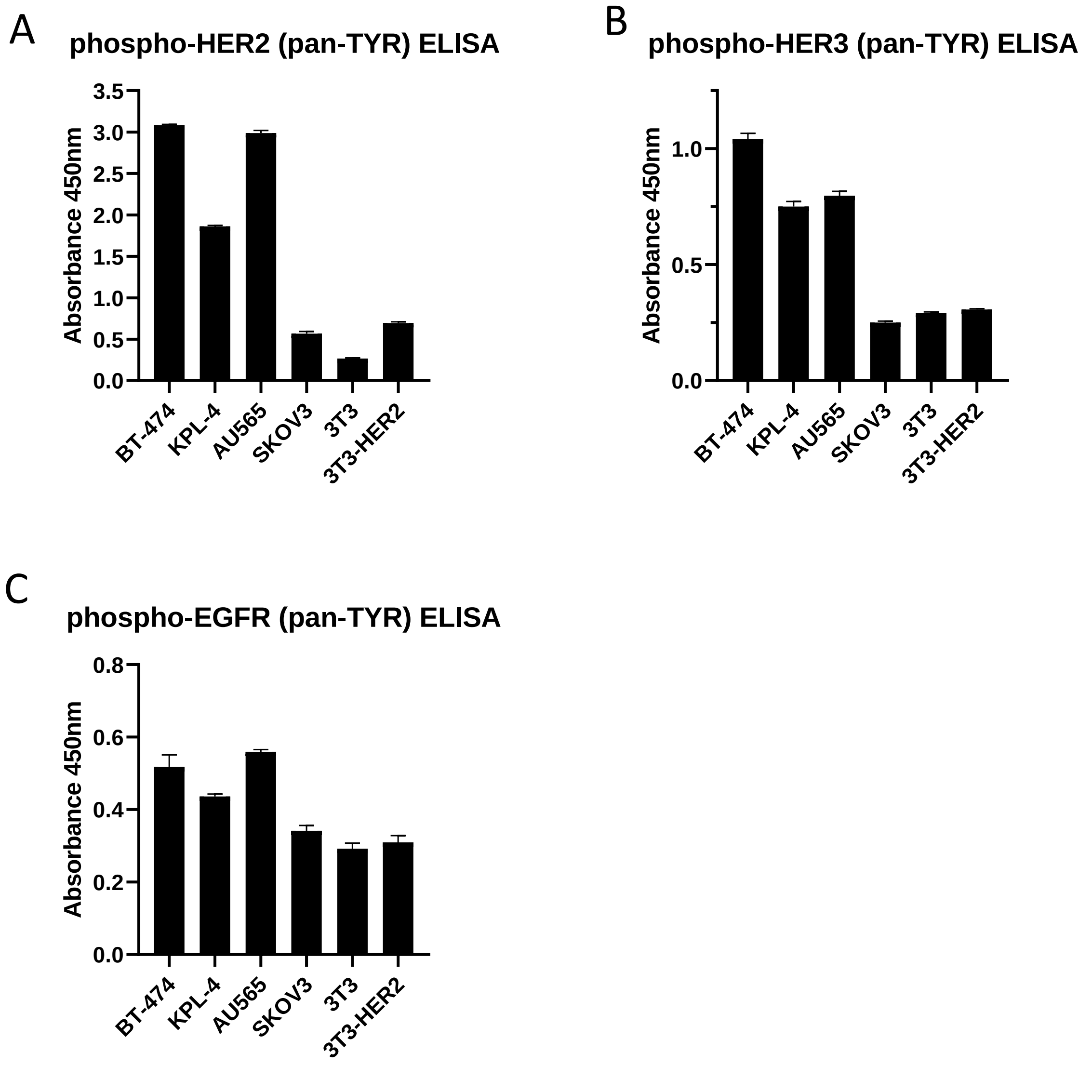

Figure S3

Tyrosine phosphorylation levels of **(A)** HER2, **(B)** HER3 and **(C)** EGFR were detected and quantified using PathScan Phospho-ErbB family (pan-Tyrosine) Sandwich ELISA assay.

Supplementary Figure 4

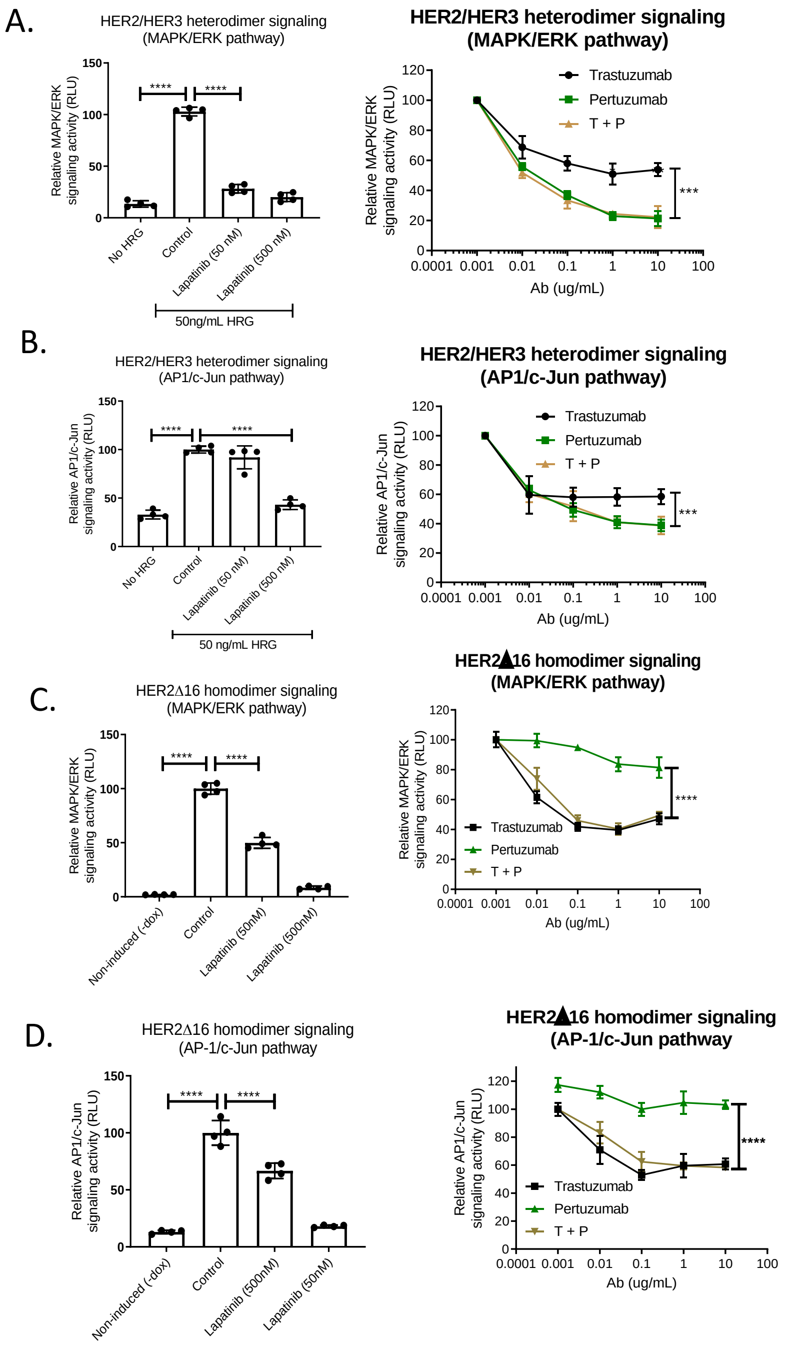

Figure S4

(A-B) HER2/HER3 heterodimer signaling assays were performed using 293T cells stably expressing HER2 and HER3. Cells were transfected with luciferase reporter constructs for (A) MAPK/ERK or (B) AP-1/c-JUN pathways and after 24h cells were treated with Heregulin β-1 (50 ng/mL). Trastuzumab and Pertuzumab were added at indicated concentrations to inhibit HER2 signaling. HER2-Tyrosine kinase inhibitor Lapatinib was used as positive assay control at the highest possible dose (500nM) without inducing cell toxicity. HER2/HER3 signaling activity were measured by luciferase readout after 24h of treatments. Mean ±SEM , n=4. two-sided t-test. **(C-D)** HER2 homodimer signaling assays were performed using doxycycline-inducible expression of HER2Δ16 in 293T cells. Cells were transfected with luciferase reporter constructs for (A) MAPK/ERK or (B) AP-1/c-JUN pathways and also treated with doxycycline for HER2Δ16 expression for 24h. Then cells were treated with HER2 mAbs at the indicated concentrations to inhibit HER2 signaling. Lapatinib was used as positive assay control. HER2 homodimer signaling activity were measured by luciferase readout after 24h of treatments. Mean ±SEM , n=4. two-sided t-test.

Supplementary Figure 5

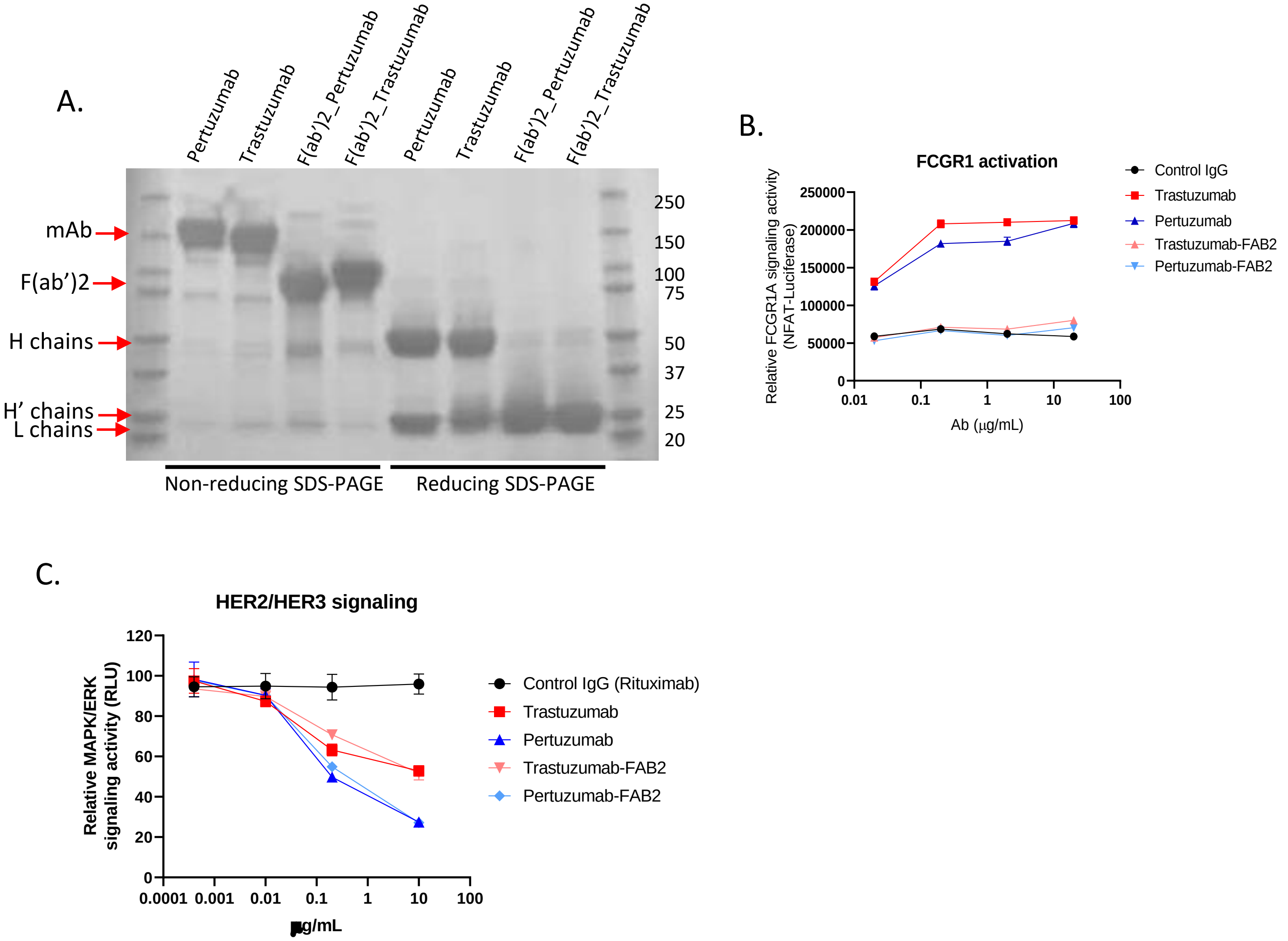

Figure S5

(A) Antigen-binding-fragment F(ab')2 fragments of Trastuzumab and Pertuzumab antibodies were generated through Pepsin digestion. Purified F(ab')2 fragments as well as the parental IgGs were checked for their predicted size on non-reducing SDS-PAGE. Similarly, their predicted heavy and light chains were observed on reducing SDS-PAGE. **(B)** Human FCGR1 signaling activation assay as described in Figure 2 was performed for the F(ab')2 fragments of Trastuzumab and Pertuzumab, and compared to their parental antibodies. (**C)** HER2/HER3 heterodimer signaling inhibition assay as described in Figure S1 was performed for the F(ab')2 fragments of Trastuzumab and Pertuzumab, and compared to their parental antibodies.

Supplementary Figure 6

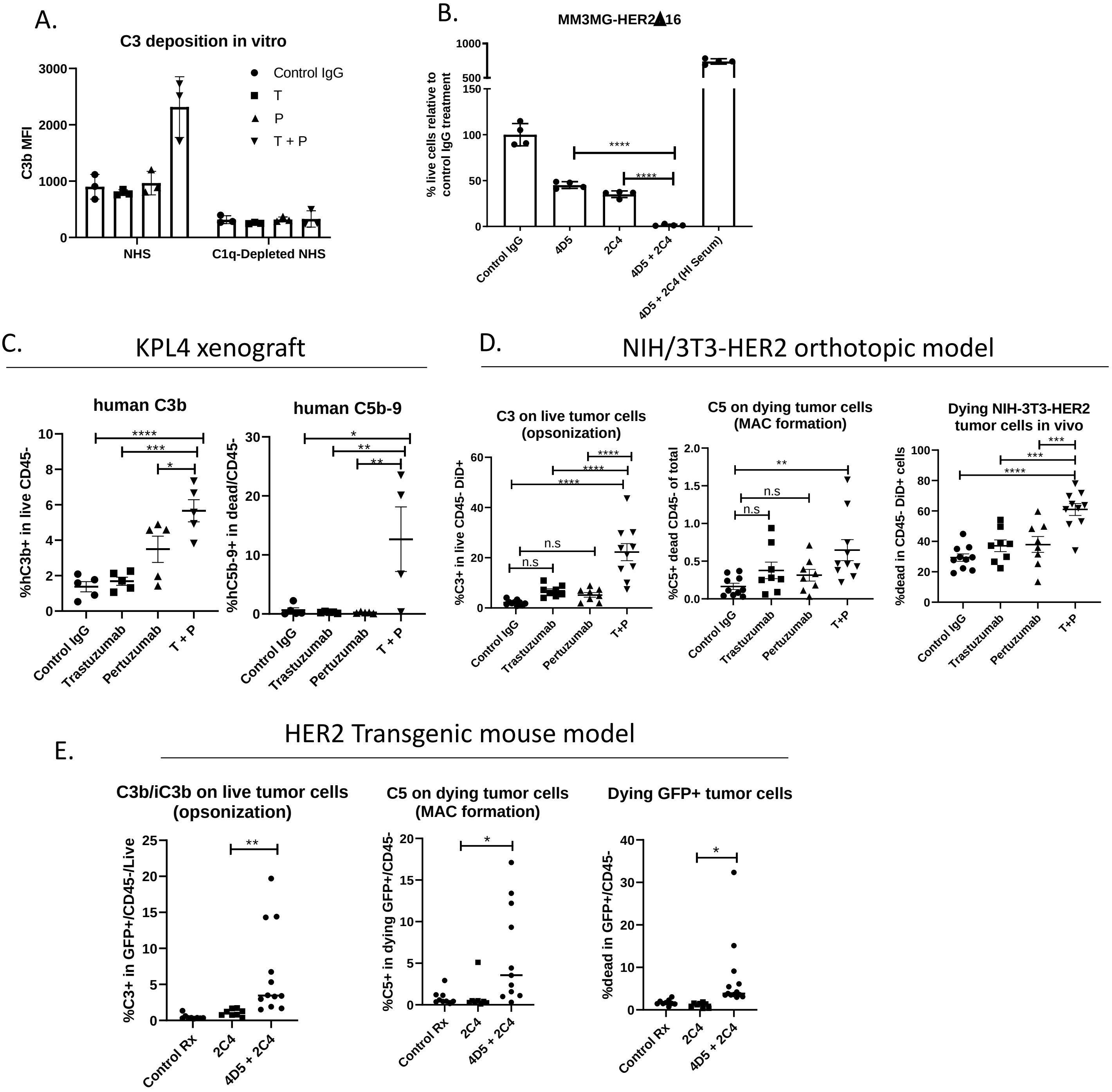

Figure S6

(A) C3b deposition quantification on KPL-4 cells as described in Figure 3A-B was repeated using C1q-depleted normal human serum. n=3 **(B)** Complement-dependent-cytotoxicity (CDC) killing assays as described in Figure 3C was repeated using murinized HER2 mAbs (4D5-IgG2A and 2C4-IgG2A) on MM3MG-HER2Δ16 target cells. n=4. **(C)** Harvested tumors from experiment described in Figure 4 were stained for human C3b and human C5b-9 antibodies and analyzed by flow cytometry. **(D)** Similar in vivo complement activation analysis described in Figure 4 was repeated using DiD-labeled NIH/3T3-HER2 cells implanted into flank of SCID-Beige mice. **(E)** In vivo complement activation analysis (C3, C5, tumor cell death) was performed on tumors harvested from HERΔ16 transgenic mice treated with murinized HER2 mAbs (Figure 1E). Tumor cells were identified by the expression of the GFP transgene. C3+, C5+ and tumor killing quantification were gated on GFP+ CD45- cells. All data analyzed by One-way ANOVA test with Tukey's multiple comparisons. Mean ±SEM, $*P<0.05$, $**P<0.01$, $***P < 0.001$, $****P<0.0001$.

Supplementary Figure 7

A.

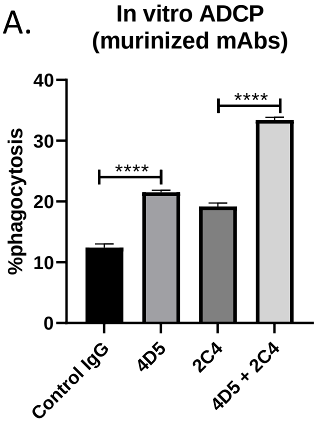

B.

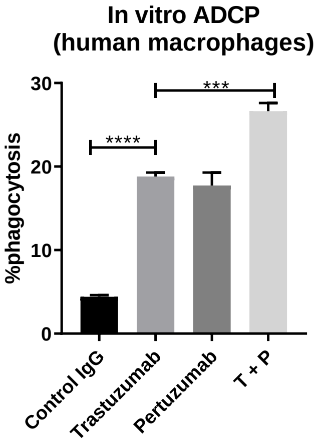

C.

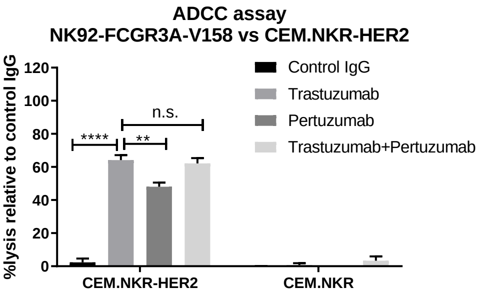

Figure S7

(A) Similar ADCP experiment as described in Figure 5A was repeated using MM3MG-HER2 cells treated with murinized HER2 mAbs (4D5-IgG2a and 2C4-IgG2A), and co-cultured with mouse bone-marrow-derived-macrophages (BMDM). Mean ±SEM , n=4. **(B)** ADCP assay as described in Figure 5A using HER2 mAbs against KPL-4 cells were repeated using human monocytes-derived macrophages (hMDM). Experiment results were repeated from three separate PBMC donors. **(C)** ADCC induction by NK cells and HER2 mAbs were measured using NK.92 cells expressing human FCGR3A-V158. Luciferase-expressing CEM.NKR-HER2 cells were used as target cells, whereas parental CEM-NKR cells were used as negative control targets. % ADCC-mediated lysis of target cells after HER2 mAbs treatment were measured by reduction of luciferase levels relative to control IgG treatment

Supplementary Figure 8

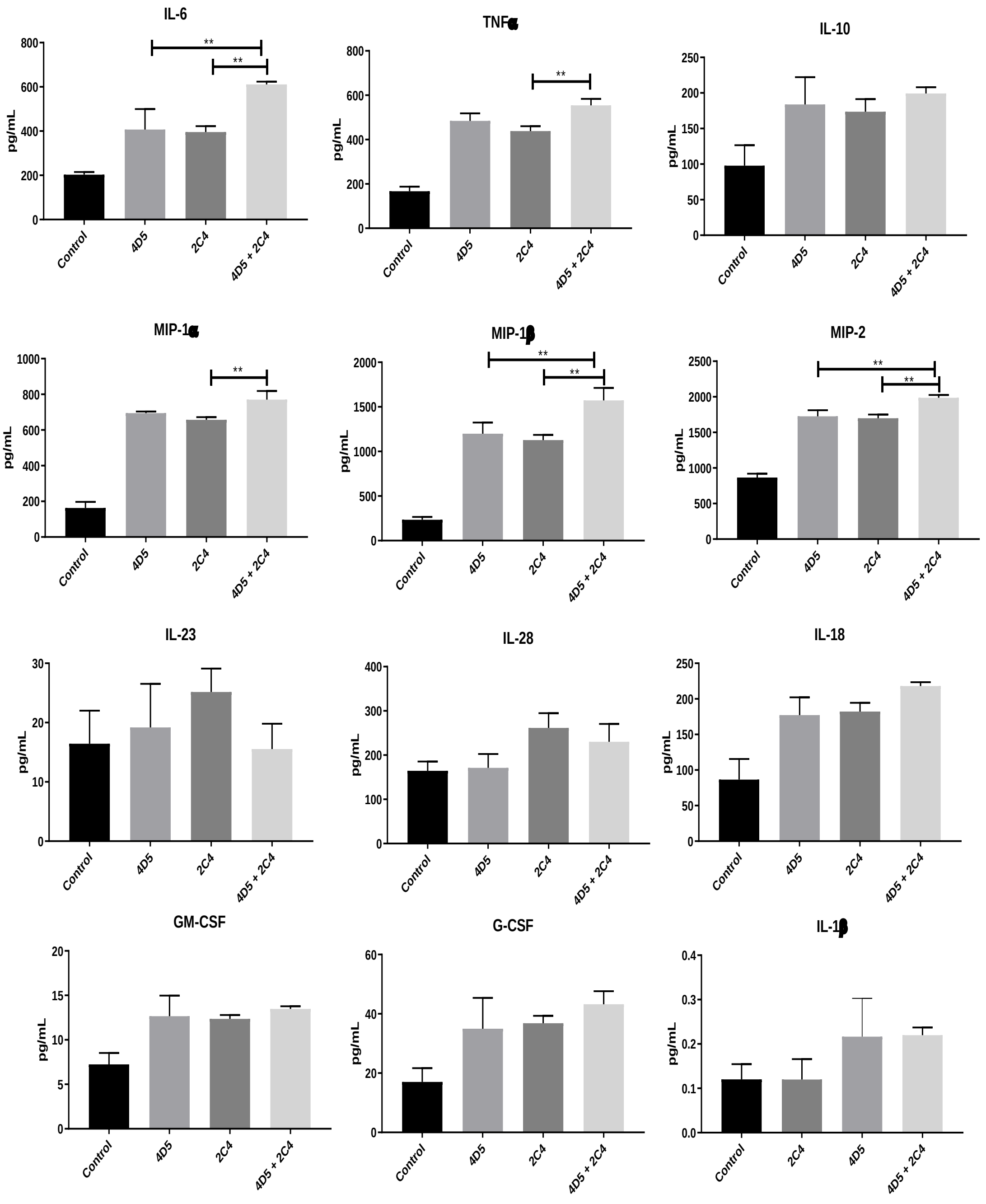

Figure S8

Secreted cytokines and chemokines by mouse bone-marrow-derived macrophages from co-culture ADCP experiment with HER2+ BC treated with murinized Trastuzumab (4D5-IgG2A), Pertuzumab (2C4-IgG2A) or their combination (4D5 + 2C4) were analyzed using the Luminex platform. *n* = 3, One-way ANOVA test with Tukey's multiple comparisons. All data represent mean ±SEM, *P<0.05, **P<0.01.

Supplementary Figure 9

A.

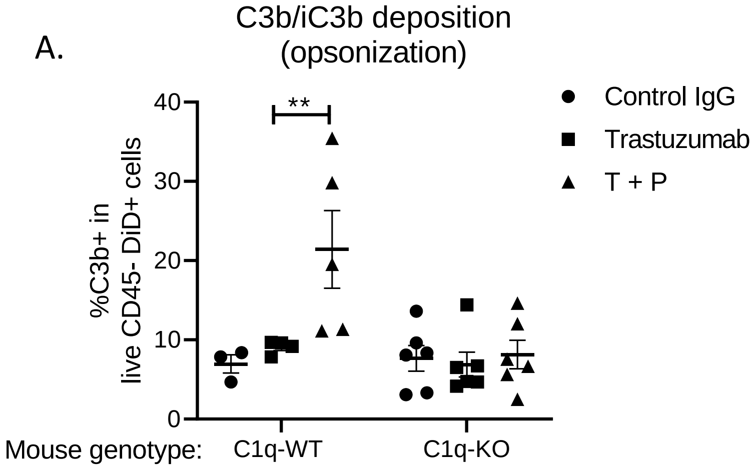

B.

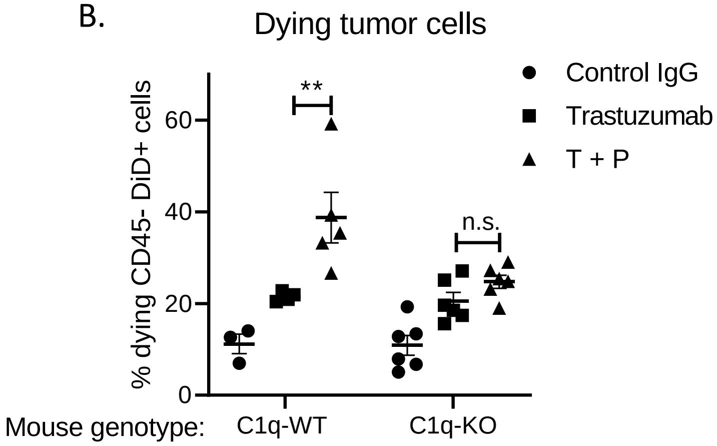

Figure S9

(A) Supplementary data to Figure 6A-B. C3b deposition on DiD-labeled KPL-4 tumors implanted in C1q$^{-/-}$ and C1q$^{+/+}$ mice were analyzed by flow cytometry after treatment with HER2 mAbs. **(B)** Dying tumor cells were also analyzed in the same experiment.

Supplementary Figure 10

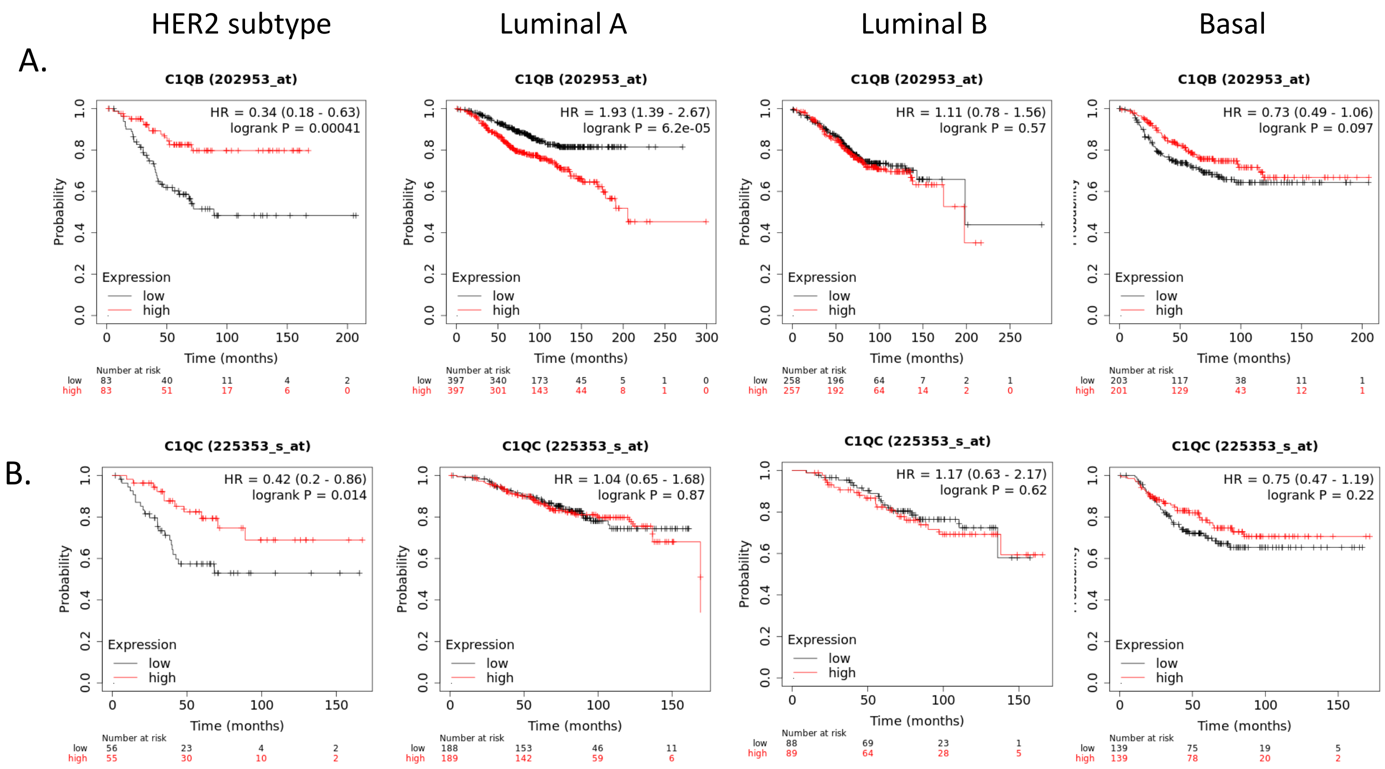

Figure S10

(A-B) The prognostic value of *C1QB* and *C1QC* expression in breast cancer patients. BC patients in KM Plotter database were classified into HER2+, Luminal A, Luminal B and Basal subtypes using St. Gallen classification. For each subtype, patients were split into two groups by their expression levels of *C1QB* or *C1QC*, and the overall survival (OS) were compared between patients with high and low gene expression.

Supplementary Figure 11

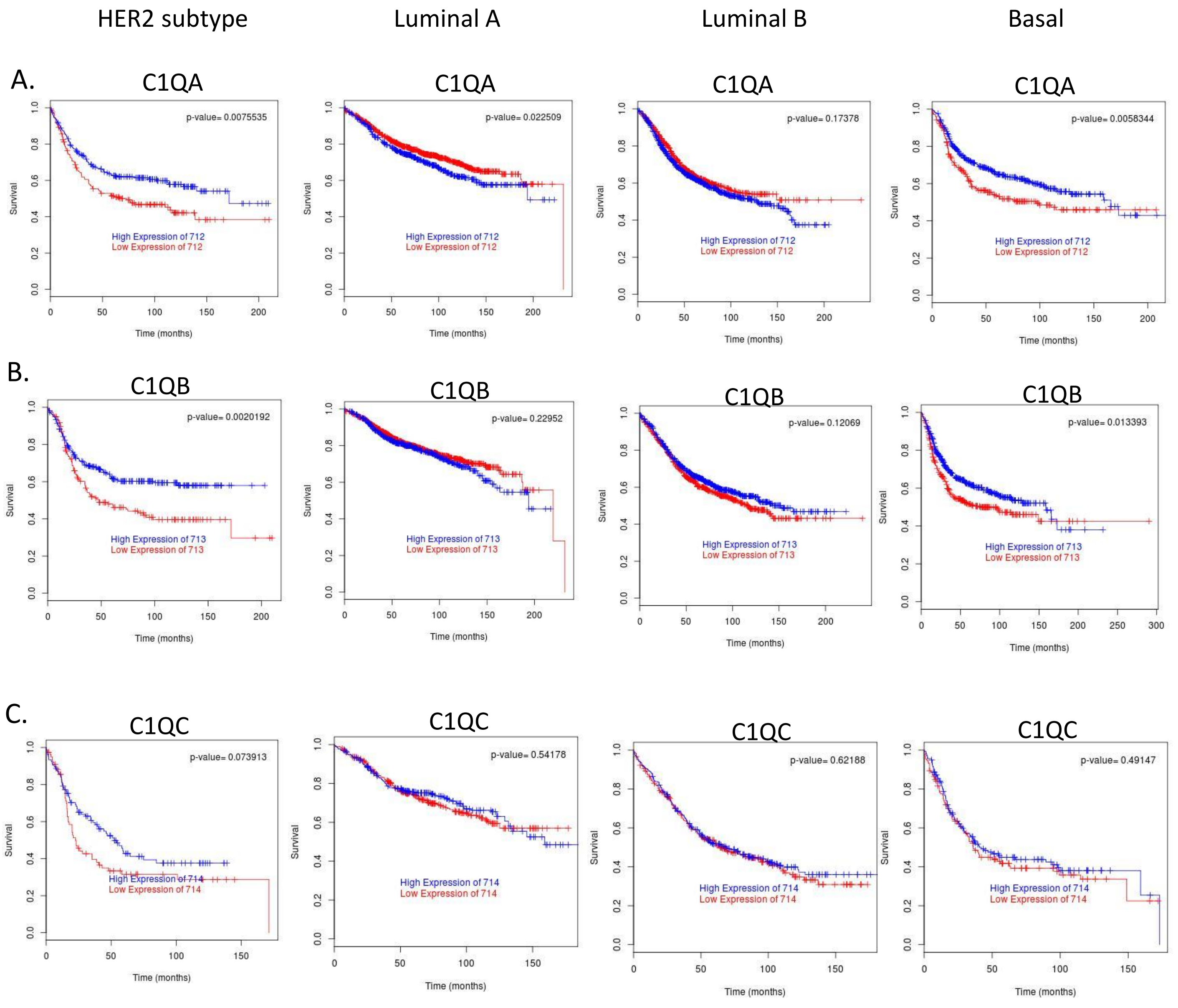

Figure S11
(A, B, C) The prognostic value of *C1QA, C1QB* and *C1QC* expression in breast cancer patients in the BreastMark dataset. Breast cancer patients were classified into HER2+, Luminal A, Luminal B and Basal subtypes using PAM50 classification. For each subtype, patients were split into two groups by their median expression levels of each *C1q* gene, and the overall survival (OS) were compared between patients with high and low gene expression.

Supplementary Figure 12

BreastMark PAM50 dataset:
CD55+CD59 combined gene expression vs overall survival

HER2 subtype

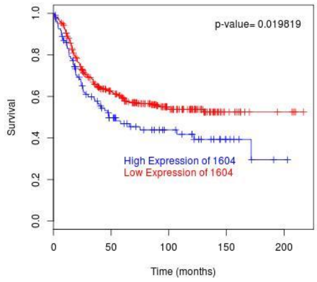

Luminal A

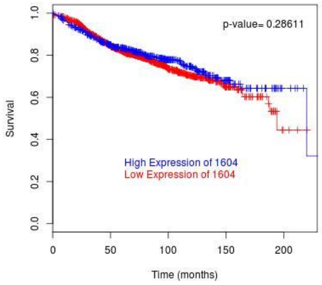

Luminal B

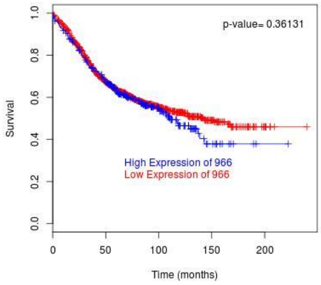

Basal

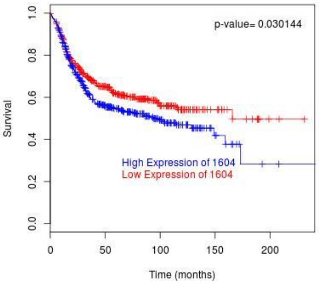

Figure S12
The prognostic value of *CD55* and *CD59* expression (combined analysis) in breast cancer patients in the BreastMark dataset. Breast cancer patients were classified into HER2+, Luminal A, Luminal B and Basal subtypes using PAM50 classification. For each subtype, patients were split into two groups by their median expression levels of *CD55* + *CD59*, and the overall survival (OS) were compared between patients with high and low gene expression.

Supplementary Figure 13

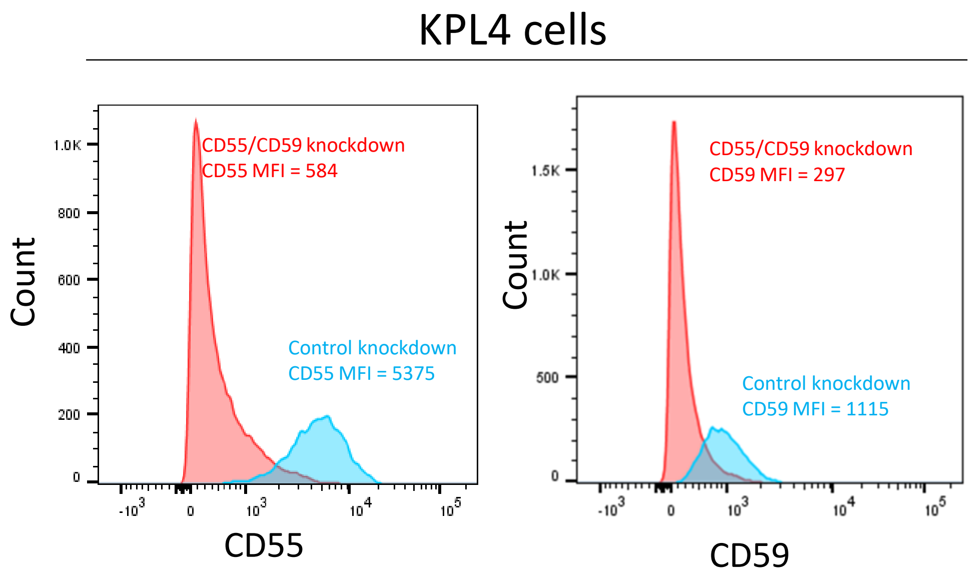

Figure S13
KPL-4 cells were stably transduced with lentivirus vector (pLKO.1) expressing shRNA against CD55 and CD59 transcripts. Empty vector transduction was used in control group. Transduced cells were selected with puromycin for 7 days and the expression of CD55 and CD59 were quantified by flow cytometry.

Supplementary Figure 14

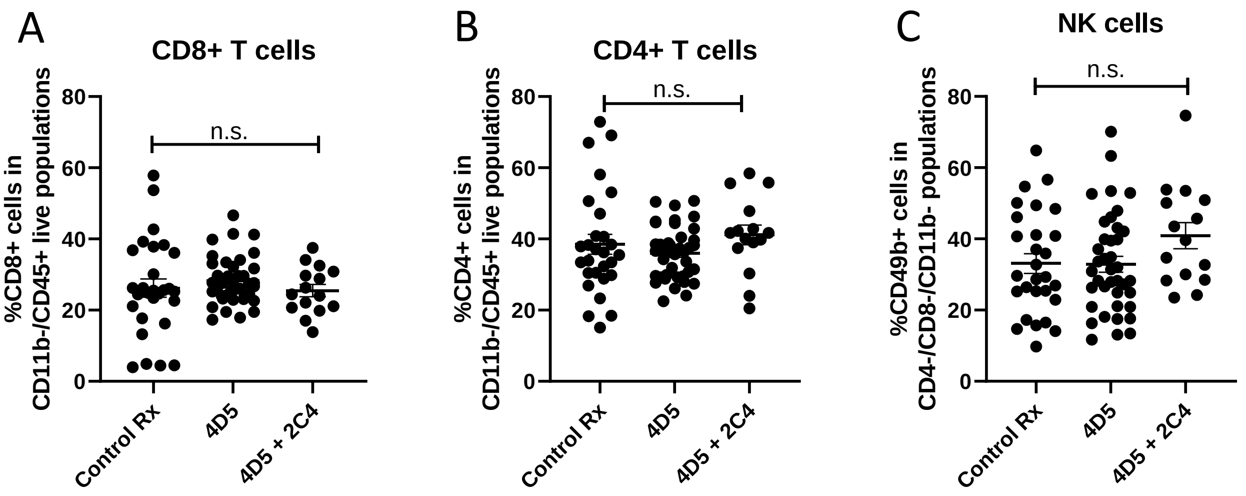

Figure S14

(A-C) Lymphoid cells compartments in tumors from the immunocompetent HER2 transgenic mice described in Figure 1E were analyzed by flow cytometry. **(D)** CD8 T cells identified as live CD45+/CD11b-/CD8+/CD4-. (E) CD4 T cells identified as live CD45+/CD11b-/CD8-/CD4+. (F) NK cells identified as live CD45+/CD11b-/CD8-/CD4-/CD49b+. All data represent mean ±SEM, *P<0.05, **P<0.01, ***P < 0.001, ****P<0.0001.

Supplementary Methods:

Western Blot

HER2-expressing cell lines (KPL-4, BT-474, Au565, SKOV3 and NIH/3T3-HER2) were lysed with Cell Lysis Buffer (CST #9803) at 1x10^7 cells per mL lysis buffer containing protease/phosphatase inhibitor (CST #5872). Western blots were performed using the LI-COR technology platform according to manufacturer's protocol. Membranes were probed overnight with antibodies against HER2 (CST #29D8), HER3 (CST # D22C5), EGFR (CST #D38B1) and GAPDH (CST #D4C6R). Secondary antibodies from LI-COR technology were used: IRDye-800CW Donkey anti-Rabbit (Cat. #926-3221) and IRDye-680RD anti-Mouse (Cat# 926-68072).

Flow cytometry quantification of HER2, HER3 and EGFR expression

HER2-expressing cell lines (KPL-4, BT-474, Au565, SKOV3 and NIH/3T3-HER2) were stained with fluorescent-conjugated antibodies for HER2 (APC-conjugated, Biolegend 324408), HER3 (PE-conjugated, Biolegend 324706) and EGFR (PB-conjugated, Biolegend 352911). Cells were stained in PBS containing 1% BSA for 15 minutes at room temperature, and analyzed on the LSRII flow cytometry machine.

Quantification of ERBB proteins phosphorylation

To detect and quantify phosphorylated forms of HER2, HER3 and EGFR in our HER2-expressing cell lines, we utilized PathScan® sandwich ELISA assay kits for Phospho-HER2 (CST #7968), Phospho-HER3 (CST #7890) and Phospho-EGFR (CST #7911). 1x10^7 cells were lysed in 1 mL lysis buffer (CST #9803) containing protease/phosphatase inhibitor (CST

#5872). A lysate concentration of 0.5 mg/mL were used for all three ELISA kits, and manufacturer's protocol were followed.

Multiplex cytokine and chemokine assay.

Mouse BMDM were co-cultured with HER2+ BC KPL-4 cells for 24 hours in the presence of murinized versions of HER2 mAbs (4D5-IgG2A and 2C4-IgG2A), and supernatants were harvested for analysis of cytokines/chemokines levels. The 26-Plex Mouse ProcartaPlex™ Panel1 kit (Thermo) was used and analyzed using the Luminex MAGPIX system.
